# Circular RNA circRHOBTB3 represses metastasis by regulating the HuR-mediated mRNA stability of PTBP1 in colorectal cancer

**DOI:** 10.7150/thno.59546

**Published:** 2021-06-01

**Authors:** Jiaxin Chen, Yizheng Wu, Xin Luo, Dongai Jin, Wei Zhou, Zhenyu Ju, Di Wang, Qing Meng, Huijuan Wang, Xiaotian Fu, Jianbin Xu, Zhangfa Song

**Affiliations:** 1 Department of Colorectal Surgery, Sir Run Run Shaw Hospital, Zhejiang University School of Medicine, 310058 Hangzhou, China.; 2 Key Laboratory of Biological Treatment of Zhejiang Province, 310058 Hangzhou, China.; 3 Department of Orthopaedic Surgery, Sir Run Run Shaw Hospital, Zhejiang University School of Medicine, 310058 Hangzhou, China.; 4 Key Laboratory of Regenerative Medicine of Ministry of Education, Institute of Aging and Regenerative Medicine, Jinan University, 510632 Guangzhou, China.

**Keywords:** circRHOBTB3, colorectal cancer, cancer metastasis, HuR, PTBP1, FUS, ADARB2

## Abstract

**Background:** Tumor metastasis of colorectal cancer (CRC) is the main cause of death in most patients and the major difficulty in comprehensive CRC treatment. Circular RNAs (circRNAs) affect many biological functions in solid tumors. However, their mechanisms in CRC metastasis remain unclear.

**Methods:** RNA sequencing (RNA-seq) and quantitative real-time PCR were performed to screen differentially expressed circRNAs between CRC tissues and adjacent normal tissues. CCK-8, cell migration and wound healing assays were performed to determine the functions of circRHOBTB3 in cell proliferation and metastasis. RNA pulldown and RNA immunoprecipitation assays were performed to verify the interaction between circRHOBTB3 and the HuR (ELAVL1) protein. Further RNA-seq and rescue experiments were applied to search for the downstream target. We also conducted a mouse xenograft model to elucidate the effect of circRHOBTB3 on cancer metastasis* in vivo*.

**Results:** We identified circRHOBTB3 which is markedly downregulated in CRC tissues and cell lines. Furthermore, lower circRHOBTB3 levels were significantly associated with advanced clinical stages and greater risk of metastases. Overexpression of circRHOBTB3 suppresses tumor metastasis in CRC cells. Mechanistically, circRHOBTB3 binds to HuR, which is a ubiquitously expressed and functional RNA-binding protein (RBP) in CRC development, and promotes β-Trcp1-mediated ubiquitination of HuR. Normally, HuR binds to the 3'UTR of target mRNAs to facilitate their stabilization, whereas the interaction between circRHOBTB3 and HuR degrades HuR to reduce the expression level of the downstream target PTBP1. Furthermore, overexpressed circRHOBTB3 suppresses lung metastases* in vivo*, and this effect can be partly reversed by PTBP1 overexpression. In addition, the transcription of circRHOBTB3 can be improved by both FUS and ADARB2 in CRC cells.

**Conclusions:** Our findings indicate that circRHOBTB3 exerts suppressive effects on CRC aggressiveness through the HuR/PTBP1 axis.

## Introduction

Colorectal cancer (CRC) is the third most frequently diagnosed cancer and the leading cause of cancer-associated death 1. Alterations at the genetic and epigenetic levels have been recognized as major players in CRC initiation and development 2. Genetic alterations include chromosomal instability (CIN), microsatellite instability (MSI), and mutations of some critical driver genes, such as Kirsten rat sarcoma viral oncogene (KRAS) and its downstream target B-Raf proto-oncogene (BRAF), adenomatous polyposis coli (APC), and tumor protein p53 (TP53) 3, 4. Epigenetic alterations mainly refer to the CpG island methylation phenotype (CIMP) 5. Following the implementation of early screening and genomic profiling to detect somatic variants, an increasing number of CRC patients have achieved early diagnosis and targeted treatment 1. However, most patients with CRC die from late-stage disease or recurrence mainly because of tumor metastasis 6. Therefore, a better understanding of the driving factors and molecular mechanisms of CRC metastasis is urgently needed to develop better treatment strategies.

Circular RNAs (circRNAs) are produced from pre-messenger RNAs (mRNAs) and pre-long noncoding RNAs (lncRNAs) through a process called back-splicing 7. Although circRNAs are generally expressed at lower levels than their linear counterparts, they can accumulate with aging because of their high stability 8. This longer half-life of circRNAs, which separates them from other RNA molecules, is probably due to the covalently closed structure that prevents circRNAs from exonuclease-regulated degradation 9. Although an increasing number of functional circRNAs have been detected through high-throughput RNA-Seq and bioinformatics algorithms in different cancers, their mechanisms have not yet been well elucidated 10. In particular, few studies have focused on the functions and underlying mechanisms of circRNAs in CRC metastasis 11.

Extensive studies have determined that circRNAs can serve as efficient microRNA (miRNA) sponges in an Argonaute (AGO2)-dependent manner 12. Encoding proteins or functional peptides independent of their parental genes is another novel mechanism by which circRNAs exert their effects 13. In addition, circRNAs function through interactions with a variety of RBPs. The patterns of circRNA-protein interactions include (i) protein scaffolds, in which circRNAs facilitate colocalization and complex formation between enzymes (such as phosphatases, transmethylases and ubiquitin ligases) and their substrates 14, 15; (ii) protein recruiters, in which specific circRNAs may recruit proteins to certain cellular locations 16, 17; (iii) protein function enhancers, in which individual circRNAs bind to and promote the functions of proteins 18; and (iv) protein sponges or decoys, in which circRNAs and other molecules with shared RBP binding motifs compete for binding with specific RBPs 19, 20. For instance, circACC1 functions as a protein scaffold to improve the enzymatic activity of the AMP-activated protein kinase (AMPK) holoenzyme by directly binding to the AMPK β and γ subunits, acting as a tumor promoter in CRC 14. Another study identified that circRNA FECR1 recruits a demethylase (TET1) to the promoter region of its host gene termed FLI1 17. Chen et al. found that N6-methyladenosine (m6A)-modified circNSUN2 acts as an enhancer of protein function by forming a ternary complex with insulin-like growth factor 2 binding protein 2 (IGF2BP2) protein and the mRNA of high motility group AT-hook 2 (HMGA2), promoting the stability of HMGA2 mRNA 18. In addition, circPTK2 functions as a protein sponge by blocking the phosphorylation sites of the vimentin protein, protecting it from phosphorylation by PKA, CDK1 or PLK 20. However, whether other specific protein-binding circRNAs can regulate CRC progression is not yet known.

In the present study, we identified hsa_circ_0007444, which is referred to as circRHOBTB3 hereafter, as a novel metastasis-related factor in CRC. The combination of fused in sarcoma (FUS) and adenosine deaminase RNA-specific B2 (ADARB2) favored the generation of circRHOBTB3. Mechanistically, circRHOBTB3 interacts with human antigen R (HuR) to promote its degradation through the ubiquitination-proteasome system (UBS). Therefore, overexpressed circRHOBTB3 attenuates the mRNA stability of the HuR target polypyrimidine tract-binding protein 1 (PTBP1) and represses PTBP1-mediated CRC metastasis.

## Materials and Methods

### Tissue samples and cell culture

A total of 83 pairs of CRC tissues and their matched adjacent nontumorous tissues was obtained from patients who underwent surgery between June 2019 and October 2020 at the Sir Run Run Shaw Hospital (Hangzhou, Zhejiang, China). The inclusion criteria of patients were as follows: primary cancer confirmed by histopathology, no preoperative chemotherapy, curative resection with lymphadenectomy and complete clinicopathologic records. At the time of surgery, all tissue specimens were immediately preserved in RNAlater (Beyotime, Shanghai, China) at -80 °C until use. Among them, 3 pairs of stage III samples were used for circRNA sequencing. The CRC stage was classified according to the 8^th^ edition of the American Joint Committee on Cancer (AJCC) tumor-node-metastasis (TNM) staging system. This study was approved and monitored by the Ethics Committee of the Sir Run Run Shaw Hospital, Zhejiang University.

A panel of CRC cell lines, including RKO, HCT116, SW480, SW620, DLD-1, Colo320, HCE8693, and HT29, and 2 normal colonic epithelial cell lines (FHC and NCM460) were purchased from the American Type Culture Collection (Manassas, VA, USA). Human embryonic kidney cells (HEK-293T) were purchased from the Cell Bank of Shanghai Academy of Chinese Sciences. The mutational status of the cell lines used in this study can be obtained from the Cancer Cell Line Encyclopedia (CCLE) database and a previous study 21. All cell lines were maintained in Dulbecco's modified essential medium (DMEM) or RPML-1640 medium (Gibco BRL, Rockville, MD) with 100 µg/mL streptomycin, 100 U/mL penicillin and 10% fetal bovine serum (FBS; Gibco, NY, USA) and incubated at 37 °C in a 5% humidified CO_2_ atmosphere. To inhibit protein synthesis or degradation, cells were treated with either cycloheximide (CHX, 50 µg/mL) for the indicated periods of time or MG132 (20 µM) for 12 h along with DMSO vehicle controls.

### RNA sequencing

Total RNA was extracted from 3 pairs of freshly frozen tissues from 3 stage III CRC patients. The inclusion criteria of these patients were as follows: both males and females, aged between 60 and 75 years, no comorbidities, and no underlying health problems or other major illnesses. The RNA amount and purity were quantified using a NanoDrop ND-1000 (NanoDrop, Wilmington, DE, USA). RNA was treated with the Ribo-Zero^TM^ rRNA Removal Kit (Illumina, San Diego, USA) to deplete ribosomal RNA (rRNA) and RNase R to remove linear RNAs, followed by cDNA library construction. Before and after the rRNA depletion and RNase R experiments, concentrations of the rRNA and liner RNA were measured using Qubit, and the downstream experiments could be performed only if the removal efficiency reached more than 90%. When analyzing the results, the ratio of rRNA and liner RNA were no more than 10%. Subsequently, paired-end sequencing on an Illumina HiSeq 4000 (LC Bio, China) was performed following the vendor's recommended protocol.

### Fluorescence *in situ* hybridization (FISH)

FAM-labeled circRHOBTB3 probes were designed and synthesized by RiboBio (Guangzhou, China). The probe signals were determined with a Fluorescent *In situ* Hybridization Kit (RiboBio, Guangzhou, China) following the manufacturer's guidelines. Images were acquired using fluorescence microscopy (Eclipse E6000; Nikon, Corporation, Tokyo, Japan). The fluorescence intensity was analyzed by Image J.

### Isolation of cancer-associated fibroblasts (CAFs) and normal fibroblasts (NFs)

CAFs and NFs were derived from fresh CRC tissues and adjacent normal tissues (at least 5 cm away from the location of cancerous tissue) obtained from 10 patients with CRC. Native fibroblasts were isolated within an hour after tissue collection. The tissues were first rinsed with 75% alcohol and D-Hanks buffer to remove residual plasma. Then, the minced tissues were digested with collagenase Ⅳ (1 mg/mL, Gibco) and hyaluronidase (0.5 mg/mL, BioFroxx) at 37 °C for 1 h. The solution was centrifuged at 1000 rpm for 5 min and washed with PBS twice. After the supernatant was aspirated completely, the remaining cells were resuspended and cultured in DMEM medium containing 10% FBS, 1% penicillin and 1% streptomycin.

### RNA interference (RNAi) and plasmid transfection

Small interfering RNAs (siRNAs) targeting the junction sites of circRHOBTB3 were obtained from RiboBio (Guangzhou, China) and transfected into the DLD-1 and SW480 cell lines with Lipofectamine RNAiMAX (Invitrogen, CA, USA). The siRNAs are listed in Table S1. The sequence of wild type (WT) or mutated (MUT) circRHOBTB3 was cloned into the lentiviral pHBLV-CMV-circ-EF1-ZsGreen-T2A-Puro vector (purchased from Hanbio Co. Ltd., Shanghai, China) to generate a WT or MUT circRHOBTB3 overexpression plasmid. The 3' and 5' circ frames contain reversed complements, which could lead to the circularization of circRHOBTB3. For stable transfection of WT and MUT circRHOBTB3 plasmids or HuR or PTBP1 overexpression plasmids (purchased from Hanbio Co. Ltd., Shanghai, China), lentivirus production and infection were performed with a Lenti-Pac HIV package kit and Lipofectamine 3000 (Invitrogen, CA, USA) according to the manufacturers' instructions. After transfection, stable HCT116 and Colo320 cells were selected with puromycin (Gibco, Grand Island, NY, USA) for several days, and the surviving cells were continuously cultured as stable cells.

### RNA extraction and quantitative real-time PCR (qRT-PCR)

Total RNA was extracted from cells or tissues using a Total RNA Kit (Omega Bio-Tek, Guangzhou, China) according to the kit instructions. For RNase R treatment, total RNA was incubated with or without 3 U/µg RNase R (Epicenter Technologies, Madison, WI, USA) for 15 min at 37 °C. Cultured cells were treated with 5 µg/mL actinomycin D (AAT Bioquest, CA, USA) or DMSO and collected at the indicated time points. After treatment with RNase R or actinomycin D, reverse transcription (RT) and quantitative PCR (qPCR) were performed using an Evo M-MLV RT Premix kit and SYBR Green Premix Pro Taq HS qPCR Kit (Accurate Biotechnology (Hunan) Co., Ltd). Typically, amplification reactions were performed in a 10 µl reaction system containing 2×SYBR Green Premix, 0.2 µM primer mixture and moderate cDNA diluted with ddH_2_O. The thermal cycling profiles for qRT-PCR included heating at 95 °C for 30 s followed by 40 cycles at 95 °C for 5 s and 60 °C for 30 s, and the reaction of cDNA copy number was monitored by quantitively analyzing fluorescence emissions using a Roche LightCycler 480 II PCR instrument (Basel, Switzerland). The threshold cycle (C_t_) represented the refraction cycle number at which a positive amplification reaction was measured. β-actin was applied as an internal standard control. Expression was quantified by the 2^-ΔΔct^ method. The primers are listed in Table S1.

### Transwell assay

Migration and invasion assays were conducted using Transwell chambers or Transwell chambers precoated with Matrigel according to the manufacturer's protocols (BD Biosciences, Bedford, MA, USA). Briefly, for the migration and invasion assays, 5×10^4^ cells were plated in 200 µl of serum-free medium on upper chambers inserted into a 24-well plate, and 600 µl of medium containing 10% FBS was added to the bottom chamber. After incubation for 24 h for the migration assay and 48 h for the invasion assay at 37 °C, nonmigrating or noninvading cells were gently removed, and the invaded cells in the lower filters were fixed with 4% polymethanol for 20 min, stained with crystal violet (Sigma, MO, USA) and counted under a microscope. The experiments were performed in triplicate.

### Wound-healing assay

Cell motility was assessed using a scratch wound assay. The transfected cells and the controls were cultured in medium containing 1% FBS in 6-well plates. When the cells reached 100% confluence, the cell monolayer was subsequently scratched with a 10 µl pipette tip. Cells were washed twice with PBS and photographed under a phase contrast microscope at different time points after wounding. The width of wound healing was quantified and compared with baseline values, and the results were expressed as the relative migration rate. Relative migration rate = migration distance/scratched width. Specifically, the migration distance equals the width that cell migrates over 24 h or 48 h. The scratched width means the distance that we scratched at 0 h. Importantly, we strictly guarantee that the scratched widths we implemented were almost the same to exclude any other interfering factors. The experiments were repeated at least three times.

### Antibodies and Western blotting

The primary antibodies used were anti-HuR antibody (11910-1-AP, Proteintech), anti-PTBP1 antibody (12582-1-AP, Proteintech), anti-RHOBTB3 antibody (13945-1-AP, Proteintech), anti-E-cadherin antibody (3195, Cell Signaling Technology), anti-N-cadherin antibody (sc-8424, Santa Cruz), anti-CD44 antibody (15675-1-AP, Proteintech), anti-MMP2 antibody (ab92536, Abcam), anti-ADAR3 antibody (sc-73410, Santa Cruz), anti-PKM1 antibody (7067, Cell Signaling Technology), anti-PKM2 antibody (4053, Cell Signaling Technology), and anti-β-actin antibody (3700, Cell Signaling Technology). CRC cells were collected, washed and lysed in RIPA lysis buffer (Beyotime, Hangzhou, China). Then, the protein concentration was determined using a BCA Protein Assay Kit (Beyotime). Cell lysates were separated by 8-12% SDS-PAGE and then transfected into PVDF membranes (Millipore, Schwalbach, Germany). The membrane was blocked with TBST buffer containing 5% skim milk powder and incubated with the corresponding primary antibodies at 4 °C overnight. The membrane was hybridized with an HRP-conjugated secondary antibody (FDM007 and FDR007, Fudebio, Hangzhou, China) at room temperature for 1 h. The signals were detected using an enhanced chemiluminescence kit (FD8030, Fudebio, Hangzhou, China).

### RNA pulldown and RNA-protein immunoprecipitation (RIP)

The biotin-coupled RNA complex was pulled down using a magnetic RNA-protein pulldown kit (Thermo, Waltham, MA, USA) according to the manufacturer's protocol. The 5' biotin-labeled oligonucleotide probe targeting the junction site of circRHOBTB3 was synthesized by GenePharma as follows: 5'-AGGCATTTTTTCTTTCCTGGTGTTTTTA-3'. The biotinylated circRHOBTB3 was captured with streptavidin magnetic beads and incubated with the cell lysates at 4 °C overnight. Then, the mixture was washed and eluted. The bound proteins were analyzed by Western blotting.

RIP for HuR protein was performed with a Magna RNA-binding Protein Immunoprecipitation Kit (Millipore, Bedford, MA, USA) according to the manufacturer's protocol. HCT116 or HEK-293T whole-cell lysates were prepared and incubated with 5 µg of anti-HuR antibody or anti-FLAG/DYKDDDDK (20543-1-AP, Proteintech) antibody controlled by normal rabbit IgG (A01008, GenScript) at 4 °C overnight. After treatment with proteinase K buffer, the immunoprecipitated RNAs were extracted using the RNeasy MinElute Cleanup Kit (Qiagen, Duesseldorf, Germany). Then, the relative expression of circRHOBTB3 was detected by qRT-PCR and normalized to input samples.

### Immunoprecipitation (IP) assay

Anti-HuR antibody was used in the IP ubiquitination assay. MG132-treated HCT116 cells were collected and incubated with Protein-A/G MagBeads (Yeason, Shanghai, China) and antibody. After overnight incubation, the immune complexes were centrifuged and washed, and the proteins were detected by Western blotting with an anti-ubiquitin antibody (3936, Cell Signaling Technology) according to the manufacturer's protocol. Anti-HuR and anti-βTrCP (4394, Cell Signaling Technology) antibodies were used in the Co-IP assays.

### Animal model

For the lung metastasis model, 2×10^6^ HCT116 cells less than 15 passages after the initial transfection were suspended in sterile PBS and injected intravenously into the tail vein of each BALB/c nude mouse (male, 4 weeks old). After five weeks, the mice were euthanatized. To image the tumors in live animals, the mice were anesthetized using isoflurane and intraperitoneally injected with 150 mg/kg D-luciferin (Yeason, Shanghai, China). After 15 min, tumor cells labeled by luminescence were imaged using a Spectrum *in vivo* imaging system (IVIS) (PerkinElmer, USA). The lung tissues were harvested and fixed with phosphate-buffered formalin for further hematoxylin and eosin (H&E) and immunohistochemistry (IHC) staining according to the manufacturer's protocol.

### Statistical analysis

To analyze the data of circRNA sequencing, Cutadapt was firstly used to remove the reads that contained adaptor contamination, low-quality bases and undetermined bases. Then, sequence quality was verified using FastQC. Bowtie2 22 and TopHat2 23 were used to map reads to the genome of species. The remaining reads (unmapped reads) were still mapped to the genome using TopHat-fusion 24. Then, we used CIRCexplorer2 25, 26 and CIRI 27 to *de novo* assemble the mapped reads to circRNAs. Moreover, back-splicing reads were identified in unmapped reads by TopHat fusion. All samples generated unique circRNAs. CircRNA expression from different samples or groups was calculated by scripts in house. Only comparisons with a P value less than 0.05 were regarded as showing differential expression by the R package 28.

The data in this study are presented as the means ± SDs. Paired or unpaired two-tailed Student's t-tests and one-way ANOVA followed by Bonferroni tests were used for the comparison of significant differences between groups with GraphPad Prism 8 software. The clinicopathological characteristics of CRC patients were analyzed with one-way ANOVA or chi-square calculations. Correlations between the circRHOBTB3 levels and ADARB2 and PTBP1 expression were analyzed by Pearson's correlation analysis. Statistical significance was set at *p < 0.05, **p < 0.01 and ***p < 0.001.

## Results

### Identification of circRHOBTB3 via RNA-Seq in CRC Tissues

**Figure 1:**
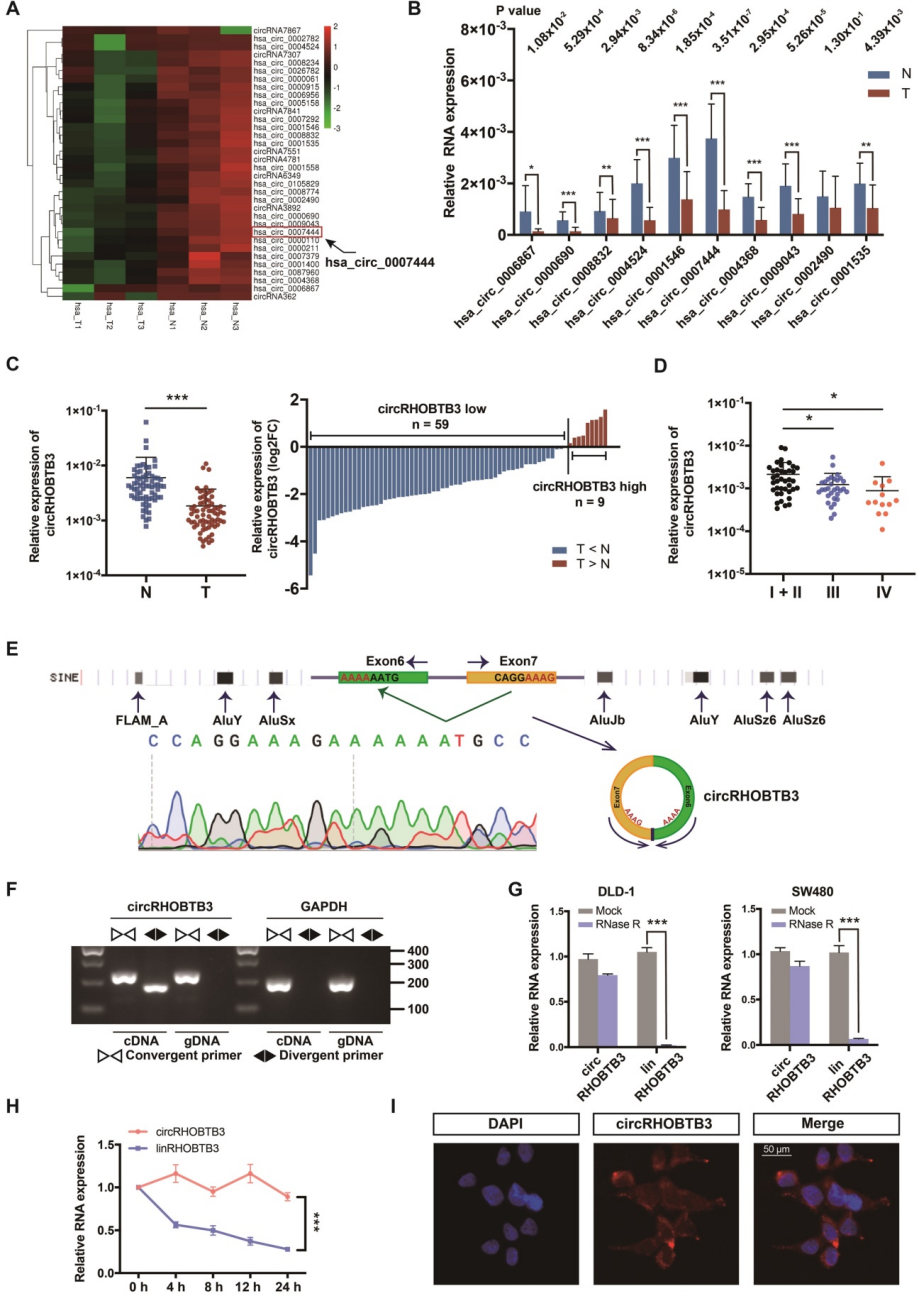
** Identification and characterization of circRHOBTB3 in CRC tissues. (A)** Clustered heatmap showing significantly downregulated circRNAs in CRC tissues compared with paired normal tissues (n = 6). The arrow indicates circRHOBTB3 (hsa_circ_0007444). **(B)** qRT-PCR validation of 10 differentially expressed circRNAs in 15 pairs of CRC samples. The P values were determined by paired Student's t-tests. The P values are shown.** (C)** qRT-PCR analysis of circRHOBTB3 expression in another 68 CRC and matched adjacent normal tissues (cases: downregulated vs. upregulated = 59 vs. 9). The P values were determined by paired Student's t-tests.** (D)** qRT-PCR analysis of circRHOBTB3 expression in 83 CRC tissues of different clinical stages. The P values were determined by one-way ANOVA. **(E)** Schematic illustration demonstrating the structure of circRHOBTB3 and Alu elements in the flanking sequence. **(F)** RT-PCR identified the presence of circRHOBTB3 in the DLD-1 cell line. Divergent primers amplified circRHOBTB3 in cDNA but not in genomic DNA. GAPDH was used as a negative control. **(G and H)** qRT-PCR detected the expression of circRHOBTB3 and linRHOBTB3 in CRC cell lines with or without RNase R or actinomycin D treatment. The P values in **H** were determined by two-way ANOVA. **(I)** FISH assay identifying the subcellular location of circRHOBTB3 in the DLD-1 cell line. Scale bar, 50 µm. The data are presented as the mean ± SD of three independent experiments, two-tailed Student's t-tests, *p < 0.05, **p < 0.01 and ***p < 0.001.

To identify potential circRNA candidates that can regulate CRC progression, we used secondary sequencing to profile differentially expressed circRNAs in 3 paired CRC and adjacent normal mucosa tissues. Notably, these tissue samples were all from stage III CRC patients. As a result, in total, 496 circRNAs were differentially expressed according to fold-change (FC) filtering (|log2FC|>1) and p value <0.05. Among them, 403 circRNAs were significantly downregulated in CRC tissues compared with normal mucosa; however, only 93 circRNAs were significantly upregulated with relatively low expression abundance in both tumor and normal tissues (Figure S1A). We first selected 10 candidates from circRNAs with significantly low expression according to their raw abundance signals and FCs (Figure 1A). Next, we measured their expression levels in 15 pairs of CRC tissues from patients, including 5 each in stage I or II, stage III and stage IV, by qRT-PCR assay. The results showed that 9 circRNAs exhibited statistically significant differences, among which circRHOBTB3 had the smallest p value and relatively high abundance (Figure 1B). Additionally, the expression of circRHOBTB3 was relatively low in colon cancer compared to other cancer types from MiOncoCirc, which is the first database composed primarily of circRNAs directly detected in tumor tissues (Figure S1B) 29. Tumor tissues consist of tumor cells and stromal cells, including fibroblasts, endothelial cells and inflammatory cells. CAFs are the key constituents of the tumor microenvironment 30. To evaluate the impact of CAFs on CRC tissue sequencing, we isolated primary CAFs from tumor tissues and NFs from paired adjacent normal tissues from 10 CRC patients and found that there was no significant difference in the expression of circRHOBTB3 between CAFs and NFs (Figure S1C), indicating that circRHOBTB3 is decreased in tumor cells instead of fibroblasts.

### Expression and characterization of circRHOBTB3 in CRC

The expression of circRHOBTB3 was further validated in another 68 pairs of human CRC specimens and benign tissues, and the results demonstrated that circRHOBTB3 was reduced in 59 tumor tissues compared with matched normal tissues (Figure 1C). We then detected the circRHOBTB3 level in the total 83 CRC tissues and found that the expression of circRHOBTB3 was significantly lower in advanced stages (stage III and stage IV) than in early stages (stage I + II), which indicated that circRHOBTB3 was negatively correlated with advanced disease staging (Figure 1D). The clinicopathological characteristics of these patients are shown in Figure S1D. To analyze the correlation between circRHOBTB3 expression in CRC tissues and clinicopathological features, the 83 patients were stratified into low and high groups based on the median value of the circRHOBTB3 expression level. As shown in Table 1, a low level of circRHOBTB3 was significantly correlated with the clinical stage, lymph node metastasis and nerve invasion. In addition, circRHOBTB3 levels were negatively correlated with the tumor size. We further evaluated the expression of circRHOBTB3 in 83 CRC patients with different clinical characteristics and stages and found that lower circRHOBTB3 levels were strongly associated with node metastasis and advanced stages (Figure S1E and G). Receiver operating characteristic (ROC) curve analysis demonstrated that circRHOBTB3 was indicative of advanced-stage CRC patients (Figure S1F-G). These collective data suggested that downregulated circRHOBTB3 might be associated with poor clinical features, including metastasis, in CRC patients.

CircRHOBTB3 is generated by back-splicing of exons 6 and 7 of the Rho-related BTB domain-containing protein 3 (RHOBTB3) gene with several Alu elements in the flanking introns on both sides (Figure 1E). We examined the expression of RHOBTB3 mRNA (linear RHOBTB3, linRHOBTB3) in 10 CRC tumor tissues and paired normal tissues but the levels were not significantly different (Figure S1H). To further confirm the characterization of circRHOBTB3, we examined the head-to-tail splicing of circRHOBTB3 by RT-PCR with convergent primers for linRHOBTB3 and divergent primers for circRHOBTB3. The results showed that circRHOBTB3 could only be detected in cDNA but not genomic DNA (gDNA) (Figure 1F). To confirm the stability, RNase R and actinomycin D treatment were employed in the experiments, and the results revealed that circRHOBTB3 was relatively resistant to RNase R and more stable than linRHOBTB3 (Figure 1G-H). FISH showed that circRHOBTB3 was localized in both the nucleus and cytoplasm (Figure 1I).

### CircRHOBTB3 correlates with CRC metastasis *in vitro*

**Figure 2:**
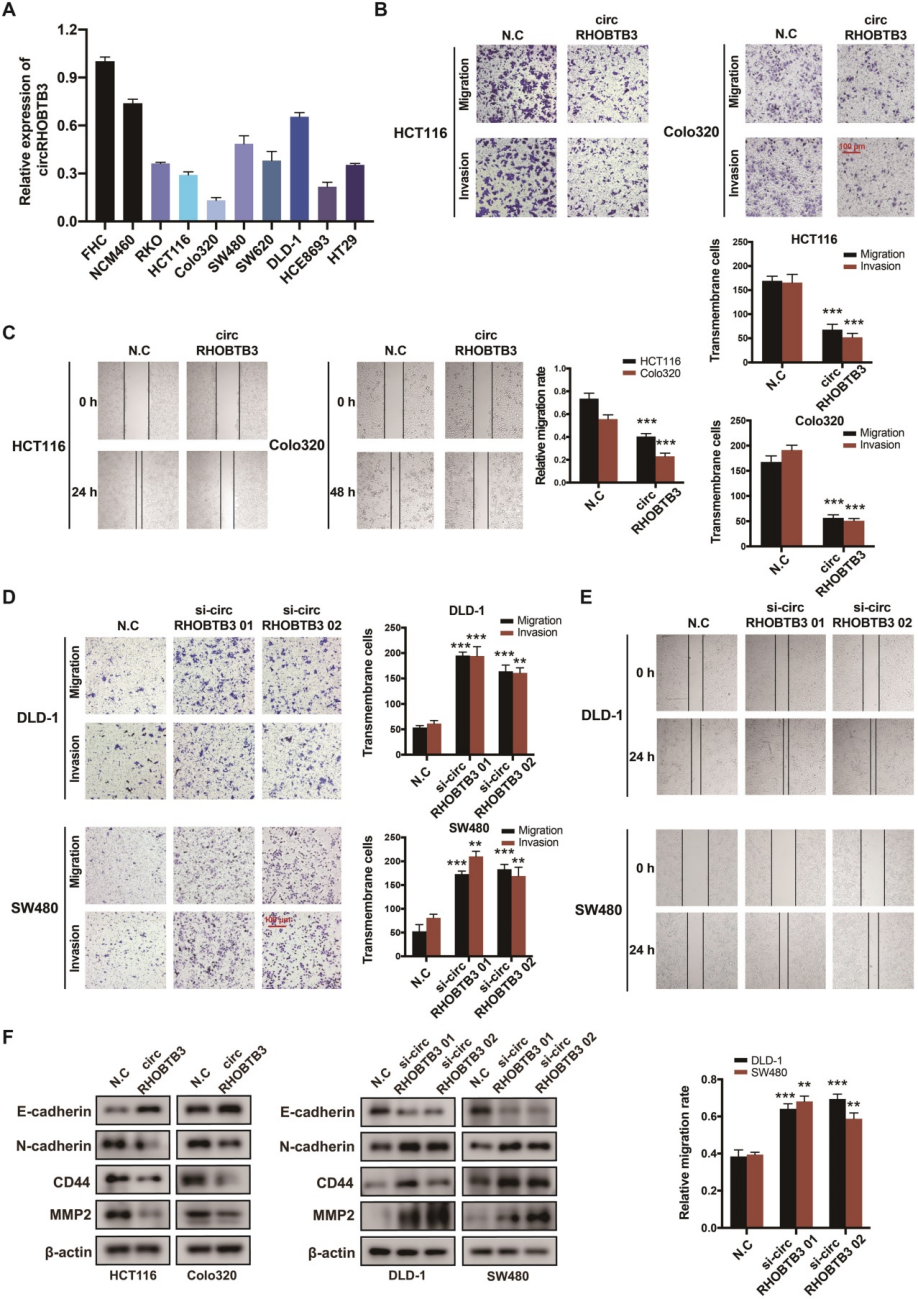
** CircRHOBTB3 inhibits CRC cell migration and invasion *in vitro*. (A)** qRT-PCR was used to determine the relative expression of circRHOBTB3 in various CRC cell lines (normalized to FHC). **(B)** Transwell migration and Matrigel invasion assays were used to assess the migration and invasion of HCT116 and Colo320 cells transfected with the N.C (control plasmid) or circRHOBTB3 plasmid (scale bar, 100 µm). **(C)** Wound healing assays measured the migration abilities of circRHOBTB3-overexpressing HCT116 and Colo320 cells. **(D)** Transwell migration and Matrigel invasion assays were used to assess the migration and invasion of DLD-1 and SW480 cells transfected with the N.C (control siRNA) or circRHOBTB3 siRNAs (scale bar, 100 µm). **(E)** Wound healing assays measured the migration capacities of circRHOBTB3-knockdown DLD-1 and SW480 cells. **(F)** Western blotting assays were applied to determine the effect of circRHOBTB3 overexpression or knockdown on the expression of EMT markers and metastasis-related proteins. The data are presented as the mean ± SD of three independent experiments, two-tailed Student's t-tests, *p < 0.05, **p < 0.01 and ***p < 0.001.

To investigate the functional role of circRHOBTB3 in CRC cells *in vitro*, we first detected its expression levels in various CRC cell lines and normal colonic epithelial cell lines, including FHC and NCM460. All CRC cells exhibited lower circRHOBTB3 expression than FHC and NCM460 cells. Among the CRC cell lines, DLD-1 and SW480 cells exhibited relatively higher levels, while HCT116 and Colo320 cells exhibited lower levels (Figure 2A). Therefore, we used these 4 cell lines in the following experiments. We transfected a circRHOBTB3 overexpression plasmid into HCT116 and Colo320 cells with high efficiency and found that it did not affect RHOBTB3 mRNA or protein levels (Figure S2A-B). CCK-8 and flow cytometry assays revealed that overexpression of circRHOBTB3 had no effects on cell proliferation (Figure S2E) or apoptosis (data not shown). However, Transwell and wound healing assays showed that increased circRHOBTB3 remarkably inhibited CRC cell migration and invasion (Figure 2B-C).

We also transfected 2 circRHOBTB3 siRNAs that significantly inhibited the expression of circRHOBTB3 but not that of RHOBTB3 mRNA and protein (Figure S2C-D). Similarly, silencing circRHOBTB3 greatly promoted malignant phenotypes (Figure 2D-E) but had no effect on cell proliferation (Figure S2F) or apoptosis (data not shown). Given the central role of epithelial-to-mesenchymal transition (EMT) in regulating cancer metastasis, we investigated whether circRHOBTB3 could mediate the EMT phenotype. Western blotting revealed that overexpression of circRHOBTB3 led to increased expression of the epithelial marker E-cadherin and decreased expression of the mesenchymal marker N-cadherin. Moreover, the expression of CRC metastasis-related proteins, including CD44 and MMP2, was markedly downregulated after circRHOBTB3 overexpression. Consistent with these results, knocking down circRHOBTB3 restrained E-cadherin expression while facilitating the expression of N-cadherin, CD44 and MMP2 (Figure 2F). Taken together, these results suggest that circRHOBTB3 has a tumor suppressive role in the context of CRC aggressiveness *in vitro*.

### CircRHOBTB3 interacts with HuR and promotes its degradation

**Figure 3:**
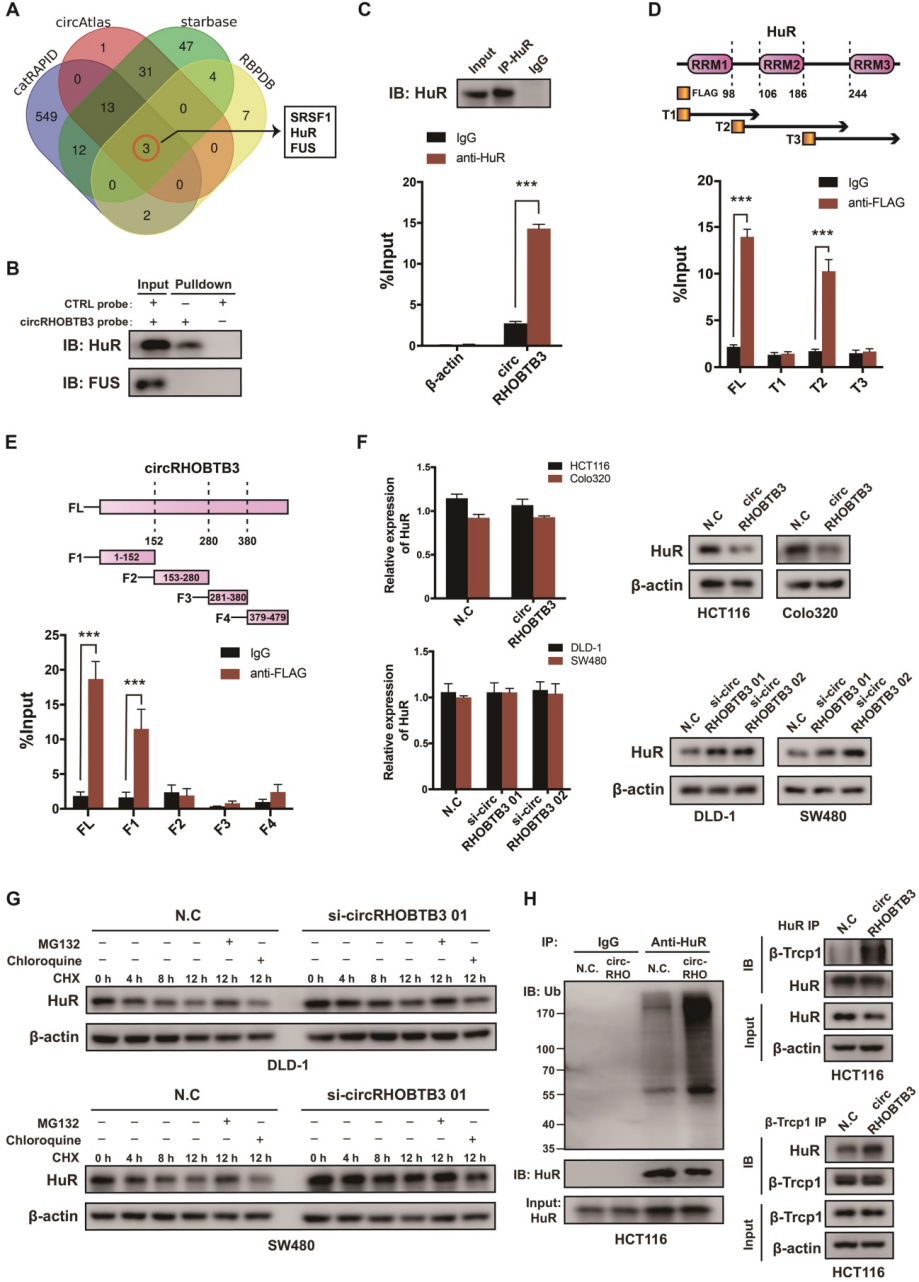
** CircRHOBTB3 associates with the HuR protein and regulates its ubiquitinated degradation in CRC. (A)** Venn diagram demonstrating the overlapping of the interacting RBPs of circRHOBTB3 predicted by catRAPID, circAtlas, starBase and RBPDB. **(B and C)** RNA pulldown and RIP experiments, respectively, confirmed the combination of circRHOBTB3 with HuR in HCT116 cells. **(D)** Schematic structures of HuR proteins and 3 truncations of HuR (top); RIP assays confirmed the interaction of truncation 2 of HuR with circRHOBTB3 in HEK-293T cells (bottom). **(E)** Schematic structures of circRHOBTB3 and 4 fragments (top); RIP assays confirmed the interaction of fragment 1 of circRHOBTB3 with HuR in HEK-293T cells (bottom). **(F)** qRT-PCR and Western blotting detected the expression of HuR mRNA and protein levels in CRC cell lines transfected with N.C, the circRHOBTB3 plasmid or siRNAs. **(G)** Western blotting detected the effect of CHX treatment with or without either MG132 or chloroquine treatment on the change in HuR protein levels mediated by circRHOBTB3 knockdown in DLD-A and SW480 cells. **(H)** After MG132 treatment, IP experiments measured the ubiquitination levels of HuR in N.C and circRHOBTB3-overexpressing HCT116 cells using an anti-HuR antibody (left); Co-IP experiments detected the interaction between HuR and the ubiquitin E3 ligase β-TrCP1 in N.C and circRHOBTB3 overexpression HCT116 cells (right). The data are presented as the mean ± SD of three independent experiments, two-tailed Student's t-tests, *p < 0.05, **p < 0.01 and ***p < 0.001.

To explore the regulatory mechanism of circRHOBTB3 in CRC metastasis, we first screened the potential circRHOBTB3-binding proteins retrieved from 4 online databases, including catRAPID, circAtlas, starBase and RBPDB (Figure 3A). According to the results, serine-rich splicing factor 1 (SRSF1), FUS and HuR could potentially interact with circRHOBTB3. Since both FUS and HuR have crucial roles in cancer progression 31-33, we then performed RNA pulldown assays using a biotin-labeled circRHOBTB3 probe, and the results verified that circRHOBTB3 could interact with HuR but not FUS from HCT116 cell extracts (Figure 3B). Further RIP assays confirmed that circRHOBTB3 was abundant in complexes precipitated by the anti-HuR antibody but not the anti-FUS antibody (data not shown) compared to those with control IgG, suggesting interaction between circRHOBTB3 and HuR (Figure 3C). HuR consists of 3 RNA recognition motifs (RRMs), and we constructed three Flag-tagged vectors encoding truncations of HuR overlapping with each other in the inactive sections. Next, HEK-293T cells were cotransfected with circRHOBTB3 and various vectors, and RIP assays demonstrated that circRHOBTB3 mainly bound to the RRM2 region (Figure 3D). Furthermore, we searched for the motifs within circRHOBTB3 that are indispensable for HuR recruitment. According to the catRAPID and MEME databases (Figure S3A-B), we constructed 4 vectors encoding circRHOBTB3 fragments and cotransfected them with Flag-tagged HuR plasmid into HEK-293T cells. RIP results indicated that fragment 1 (F1) of circRHOBTB3 interacts with HuR, which matched the predicted binding sites (Figure 3E). Next, we mutated 2 predicted binding motifs in F1 of circRHOBTB3 to determine whether the mutated circRHOBTB3 still interacted with HuR (Figure S3C). The MUT circRHOBTB3 vector was transfected into HCT116 cells and RIP assays confirmed that WT circRHOBTB3, but not MUT circRHOBTB3, interacted with HuR (Figure S3D-E).

To examine the expression correlation of circRHOBTB3 with HuR, qRT-PCR and Western blotting were performed. As a result, overexpression of circRHOBTB3 in HCT116 and Colo320 cells caused downregulation of HuR protein expression but did not decrease its mRNA level. Knockdown of circRHOBTB3 in DLD-1 and SW480 cells led to elevated levels of HuR protein but not increased mRNA levels (Figure 3F). Given these observations, we hypothesized that circRHOBTB3 may regulate the stability of the HuR protein post-transcriptionally. Thus, we treated circRHOBTB3 knockdown DLD-1 and SW480 cells with CHX to restrain protein synthesis for the indicated time periods, and the cells were cotreated with or without the proteasome inhibitor MG132 or lysosome inhibitor chloroquine. The results demonstrated that the remaining portions of HuR protein were relatively higher in circRHOBTB3-silenced cells than in N.C groups. Furthermore, the degradation of HuR could be reversed by MG132 but not chloroquine, suggesting that circRHOBTB3 likely enhanced HuR degradation through the UBS (Figure 3G). To further clarify this hypothesis, we transfected the N.C or circRHOBTB3 plasmid into HCT116 cells. After MG132 treatment, the ubiquitination of HuR was measured. As shown in Figure 3H, the extent of HuR ubiquitination was relatively improved after circRHOBTB3 overexpression compared with the N.C groups. The E3 ubiquitin ligase β-Trcp1 has been reported to specifically target HuR for ubiquitination and degradation 34; therefore, we performed Co-IP experiments and found that overexpressing circRHOBTB3 facilitated the interaction between HuR and β-Trcp1 in HCT116 cells (Figure 3H). Together, we concluded that circRHOBTB3 interacts with HuR to promote its β-Trcp1-mediated ubiquitination and degradation.

### CircRHOBTB3 degrades HuR to restrain PTBP1 expression

**Figure 4:**
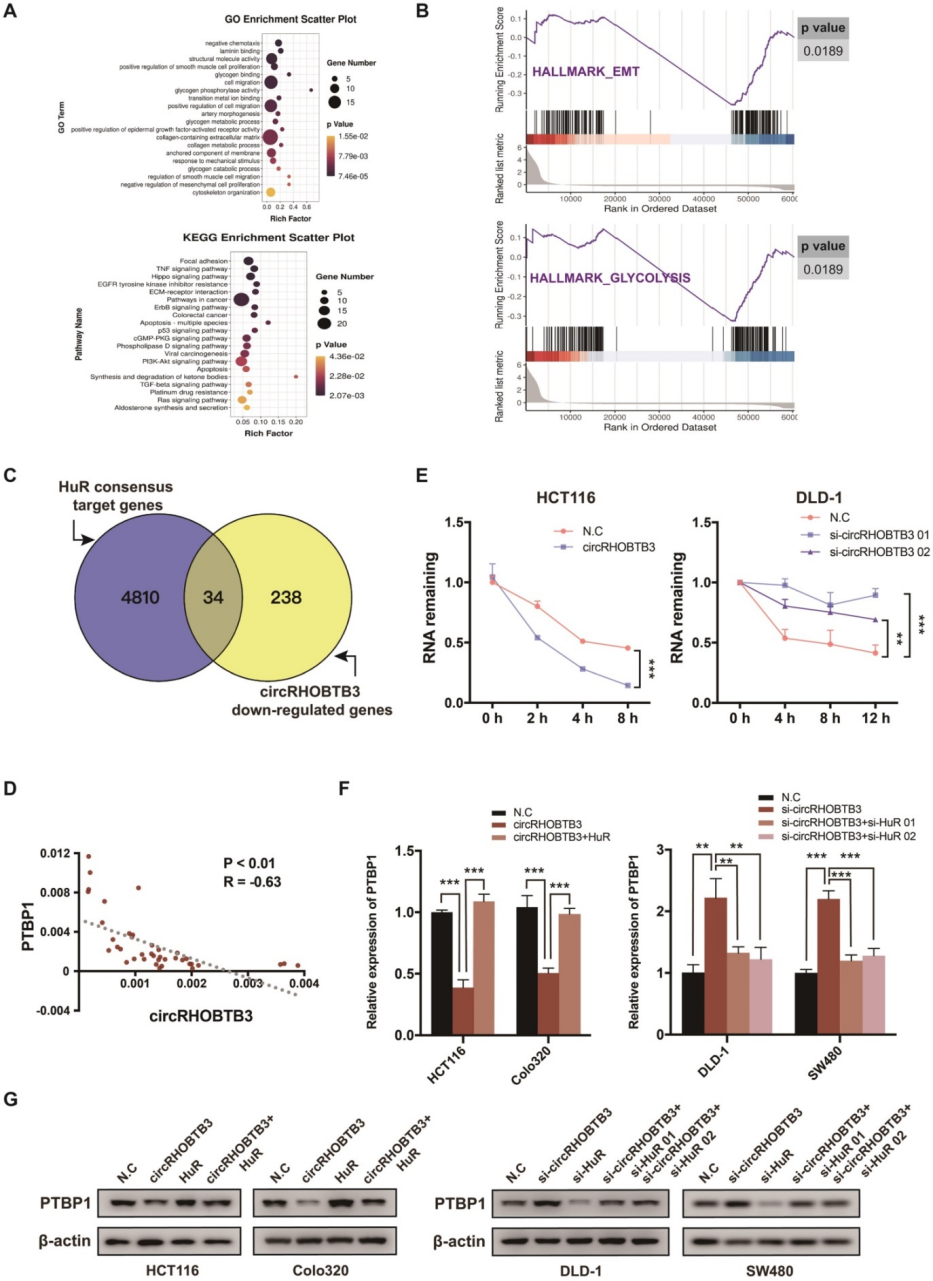
** CircRHOBTB3 degrades HuR to inhibit the expression of PTBP1. (A)** Bulb map of GO and KEGG analyses of the differentially expressed mRNAs in HCT116 cells transfected with N.C and circRHOBTB3 overexpression plasmids. **(B)** GSEA analyses showing the enrichment of genes in EMT and glycolysis. **(C)** Venn diagram exhibiting overlapping circRHOBTB3-repressed genes in the set of HuR targets determined by the previous CLIP experiment. **(D)** qRT-PCR showed a negative correlation between circRHOBTB3 and PTBP1 expression in 40 CRC tissues. The R values and P values were from Pearson's correlation analysis. **(E)** qRT-PCR estimated the influences of circRHOBTB3 on the mRNA stability of PTBP1 in HCT116 and DLD-1 cells treated with actinomycin D. The P values were determined by two-way ANOVA. **(F and G)** qRT-PCR and Western blotting analyses were adopted to test the expression of PTBP1 in N.C and circRHOBTB3-overexpressing HCT116 and Colo320 cells with or without HuR overexpression (left); qRT-PCR and Western blotting analyses showed the expression of PTBP1 in N.C and circRHOBTB3-knockdown DLD-1 and SW480 cells with or without HuR knockdown (right). The data are presented as the mean ± SD of three independent experiments, two-tailed Student's t-tests, *p < 0.05, **p < 0.01 and ***p < 0.001.

In many cancerous settings, including CRC, HuR protein exhibits increased expression and cytoplasmic abundance. This overactive status allows HuR to stabilize various mRNAs involved in CRC development 35. In this study, we speculated whether circRHOBTB3 function was mediated by certain HuR-stabilized substrates. Therefore, we screened the transcriptome by RNA-Seq, focusing on the weakly expressed genes in HCT116 cells with stable overexpression of circRHOBTB3 compared to N.C cells, which revealed 272 mRNAs that were significantly decreased (Figure S4A). Gene ontology (GO) and Kyoto Encyclopedia of Genes and Genomes (KEGG) pathway enrichment analyses showed that many enriched GO terms were associated with tumor metastasis, such as laminin binding, cell migration, cytoskeleton organization and glycogen metabolism, including glycogen binding, glycogen phosphorylation activity, glycogen metabolic and catabolic processes. According to KEGG analysis, the highly enriched pathway was focal adhesin, which is closely correlated with cell migration (Figure 4A). In addition, gene set enrichment analysis (GSEA) revealed that exogenous expression of circRHOBTB3 regulated genes involved in EMT and glycolysis in CRC (Figure 4B).

The third RRM motif of HuR is helpful for stabilizing various target mRNAs involved in tumor progression 36. Therefore, among the 272 downregulated mRNAs in our RNA-Seq, we screened HuR targets from a previous study mapping HuR-binding sites using PAR-CLIP (photoactivatable ribonucleotide enhanced crosslinking and immunoprecipitation) 37 (Figure 4C). Indeed, we found that 34 genes were both repressed by circRHOBTB3 and targeted by HuR protein and then selected 4 CRC metastasis-related candidates based on the results reported in the literature and the TCGA database (Figure S4B). After qRT-PCR and Western blotting validation, PTBP1 and CD44 were selected for further experiments (Figure S4C). As reported by Takahashi et al., PTBP1 plays a crucial role in the invasion of CRC cells, and these invasive properties arise partially through splicing CD44 38. The TCGA database also revealed that CD44 expression was positively correlated with PTBP1 (Figure S4D). Moreover, PTBP1 has been reported to mediate the pyruvate kinase (PKM) isotype switch to facilitate the Warburg effect in CRC 39. Taken together, we hypothesized that circRHOBTB3 degraded HuR to influence the expression of PTBP1 and affect tumor aggressiveness. To assess this scenario, we performed qRT-PCR in 40 cancerous tissues and found that the expression of circRHOBTB3 was negatively correlated with that of PTBP1 (Figure 4D). Moreover, overexpressed circRHOBTB3 significantly reduced the mRNA stability of PTBP1, whereas knockdown of circRHOBTB3 increased the remaining PTBP1 mRNA (Figure 4E and Figure S4E). Next, HuR was overexpressed in HCT116 and Colo320 cells or knocked down in DLD-1 and SW480 cells. The efficiency is demonstrated in Figure S4F-G. Further qRT-PCR and Western blotting assays elucidated that overexpression of HuR increased the decline in PTBP1 levels caused by circRHOBTB3 upregulation, while silencing HuR abolished the effect of circRHOBTB3 depletion on PTBP1 levels (Figure 4F-G). All of these results indicate that circRHOBTB3 interacts with HuR to prevent PTBP1 expression.

### CircRHOBTB3 inhibits PTBP1 levels to regulate cell metastasis *in vitro*

**Figure 5:**
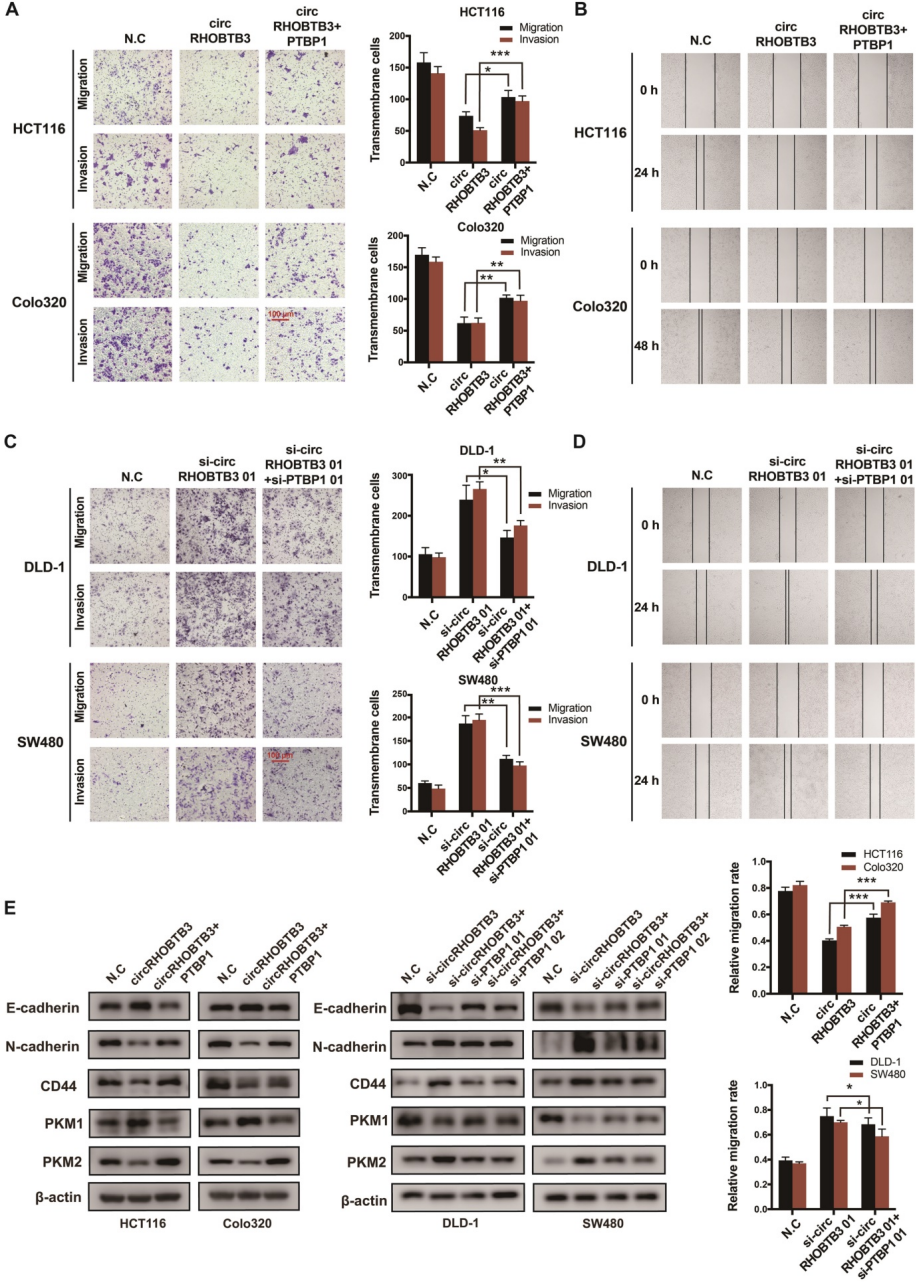
** CircRHOBTB3 retards CRC cell metastasis by mediating PTBP1. (A and B)** Transwell and wound healing assays demonstrated that the effects of circRHOBTB3 overexpression on the migration and invasion of HCT116 and Colo320 cells were partly abrogated by PTBP1 overexpression (scale bar, 100 µm). **(C and D)** Transwell and wound healing assays demonstrated that the effects of circRHOBTB3 knockdown on the migration and invasion of DLD-1 and SW480 cells were partly abrogated by PTBP1 knockdown (scale bar, 100 µm). **(E)** Western blotting assays were applied to determine the rescue abilities of PTBP1 on circRHOBTB3 overexpression or knockdown in CRC cells. The protein levels of E-cadherin, N-cadherin, CD44, and PKM1/2 were determined. The data are presented as the mean ± SD of three independent experiments, two-tailed Student's t-tests, *p < 0.05, **p < 0.01 and ***p < 0.001.

To determine whether circRHOBTB3 could regulate the PTBP1 pathway to influence CRC cell migration, PTBP1 was overexpressed in circRHOBTB3-overexpressing HCT116 and Colo320 cells or knocked down in circRHOBTB3-silenced DLD-1 and SW480 cells. The transfection efficiency was examined by qRT-PCR and Western blotting (Figure S5A-B). The first siRNAs for circRHOBTB3 and PTBP1 (si-circRHOBTB3 01 and si-PTBP1 01) with better silencing efficiencies were chosen for subsequent rescue experiments. Transwell and wound-healing assays revealed that PTBP1 overexpression was able to partly reverse the inhibitory impacts of circRHOBTB3 upregulation on cell migration and invasion (Figure 5A-B), whereas silencing PTBP1 partially restrained the acceleration of CRC aggressiveness caused by circRHOBTB3 knockdown (Figure 5C-D). Concordantly, the effect was further confirmed by detecting the expression of migration-related proteins, including E-cadherin, N-cadherin and CD44. Interestingly, we found that circRHOBTB3 expression was negatively correlated with the PKM2:PKM1 ratio in CRC cells and that PTBP1 could partly reverse this effect (Figure 5E). Altogether, these findings suggest that the suppressive function of circRHOBTB3 in CRC metastasis is regulated by inhibition of the HuR/PTBP1 axis.

### CircRHOBTB3 restricts lung metastasis *in vivo* by suppressing the PTBP1 pathway

**Figure 6:**
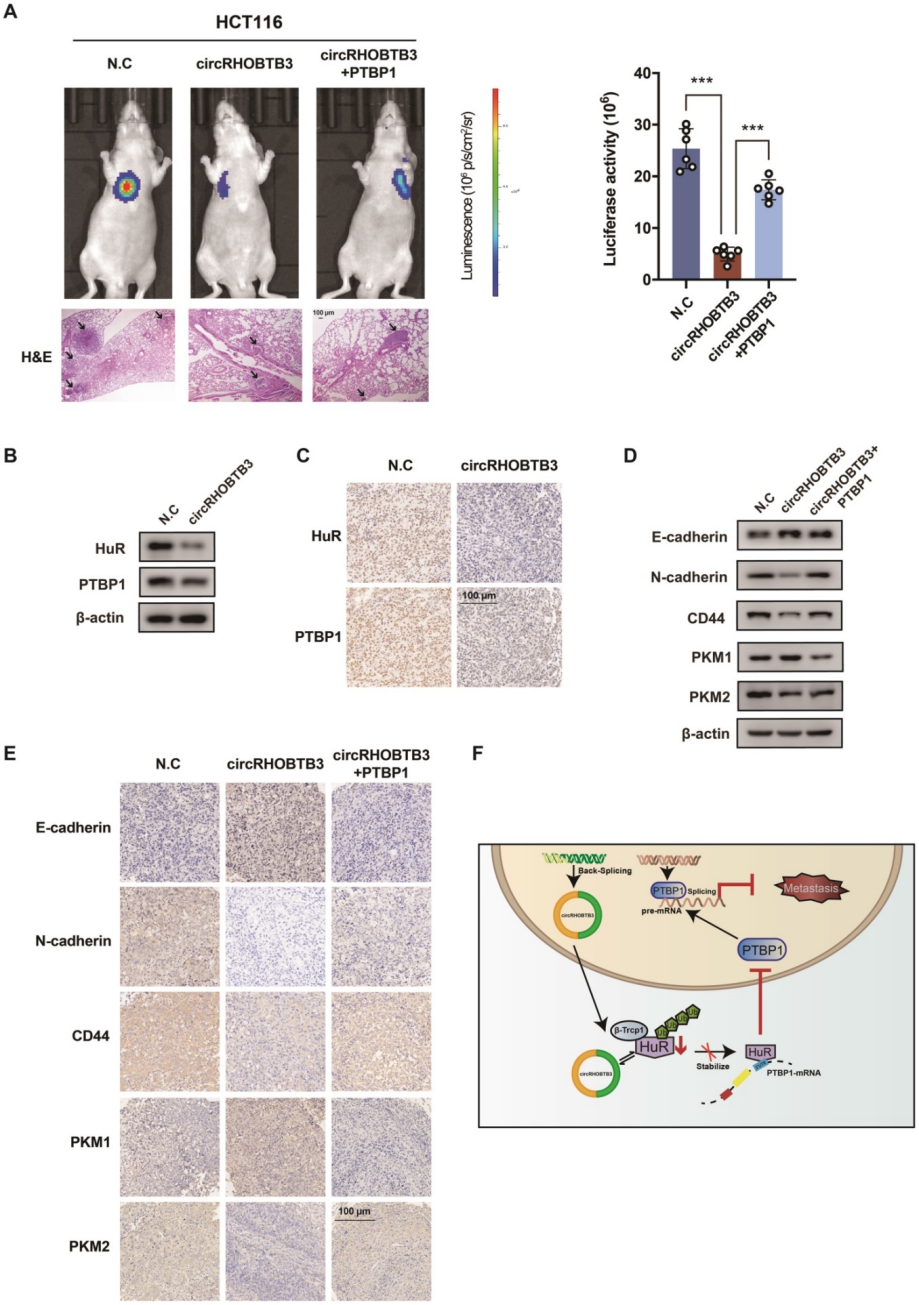
** CircRHOBTB3 inhibits CRC metastasis through the HuR/PTBP1 axis* in vivo*. (A)** Representative bioluminescence images and H&E staining (left, Scale bar, 100 µm) and a histogram (right) showed the lung metastatic lesions of mice injected with 3 different stable HCT116 cells via the tail vein (n = 6 each group). **(B and C)** Western blotting and representative IHC staining images showed the relative protein levels of HuR and PTBP1 in tumors formed by circRHOBTB3 overexpression in HCT116 cells (scale bar, 100 µm). **(D and E)** Western blotting and representative IHC staining revealed the relative protein levels of E-cadherin, N-cadherin, CD44 and PKM1/PKM2 in tumors of different groups (scale bar, 100 µm). **(F)** Schematic illustration of the regulatory landscape of the circRHOBTB3/HuR/PTBP1 axis in suppressing the metastasis of CRC. The data are presented as the mean ± SD of three independent experiments, two-tailed Student's t-tests, *p < 0.05, **p < 0.01 and ***p < 0.001.

To better map the role of circRHOBTB3 in controlling the metastasis of CRC *in vivo*, we used HCT116 cells (labeled with luminescent dye) with or without stable circRHOBTB3 overexpression or with both circRHOBTB3 and PTBP1 overexpression. Then, the cells were injected into the tail vein of nude mice for seeding into the lung cavity. *In vivo* bioluminescence imaging showed that luciferase activity was significantly decreased in the circRHOBTB3 overexpression group, and H&E staining revealed that tumor formation was markedly reduced in the circRHOBTB3 overexpression group. However, this effect was partly reversed by PTBP1 (Figure 6A). These data indicated that circRHOBTB3 inhibits cancer metastases* in vivo*. Furthermore, Western blotting and IHC staining indicated that the expression of HuR and PTBP1 was reduced after circRHOBTB3 overexpression (Figure 6B-C), which was consistent with our *in vitro* results. Subsequent Western blotting and IHC staining of the excised tumor sections demonstrated that changes in the expression of E-cadherin, N-cadherin, CD44, PKM1 and PKM2 were also in agreement with our *in vitro* results (Figure 6D-E). Taken together, the results of our study demonstrated that circRHOBTB3 promotes the ubiquitination and degradation of HuR to inhibit PTBP1 expression and CRC metastasis (Figure 6F).

### The generation of circRHOBTB3 is regulated by the combination of FUS and ADARB2

**Figure 7:**
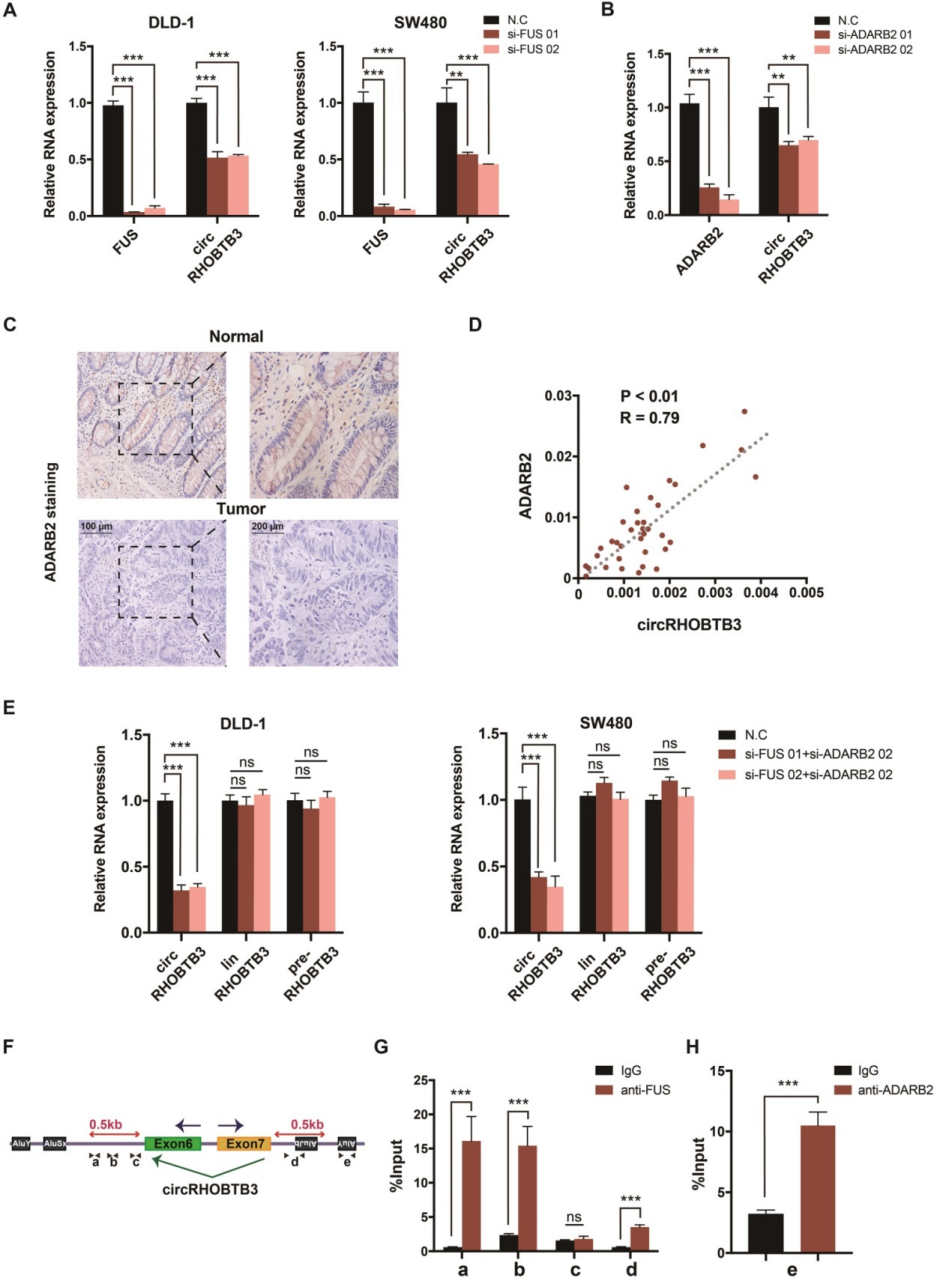
** FUS and ADARB2 promote the biogenesis of circRHOBTB3 in CRC. (A)** qRT-PCR analysis of the expression of circRHOBTB3 upon FUS depletion using siRNAs in DLD-1 and SW480 cells. **(B)** qRT-PCR analysis of the expression of circRHOBTB3 upon ADARB2 depletion using siRNAs in SW480 cells. **(C)** Representative IHC staining images showing the relative protein levels of ADARB2 in paired CRC and normal tissues (scale bars, 100 µm or 200 µm). **(D)** qRT-PCR analysis showed a positive correlation between ADARB2 and circRHOBTB3 expression in 40 CRC tissues. The R values and P values were from Pearson's correlation analysis. **(E)** qRT-PCR for the expression of circRHOBTB3, linRHOBTB3 and pre-RHOBTB3 upon the combination of FUS and ADARB2 depletion in DLD-1 and SW480 cells. **(F)** Schematic illustration of specific primers targeting the putative FUS and ADARB2-binding sites on the flanking introns of circRHOBTB3. **(G)** RIP experiments confirmed the interaction between FUS and introns within 500 nt proximal to back-splicing sites. **(H)** RIP experiments confirmed the interaction between ADARB2 and the AluY element downstream of the flanking introns of circRHOBTB3. The data are presented as the mean ± SD of three independent experiments, two-tailed Student's t-tests, *p < 0.05, **p < 0.01 and ***p < 0.001.

The generation of many circRNAs is influenced by a combination of cis-acting elements such as the Alu sequence, and trans-acting splicing factors (SFs), including various RBPs 8. To gain a deeper understanding of the role of circRHOBTB3, we further clarified which cis- or trans-acting factors can regulate circRHOBTB3 circularization. SF FUS has a well-characterized role in regulating back-splicing and circRNA formation 40. Our previous data confirmed that FUS was not able to interact with circRHOBTB3 itself; however, FUS could potentially bind to the introns flanking the circularized exons of circRHOBTB3 according to circAtlas. Therefore, we preliminarily analyzed whether FUS contributes to circRHOBTB3 biogenesis by disrupting FUS expression using 2 siRNAs (Figure S5D). Interestingly, the results showed that silencing FUS strongly suppressed the expression of circRHOBTB3 in both DLD-1 and SW480 cell lines (Figure 7A). As SFs have been shown to regulate cell-type-specific circRNA formation 41, we then retrieved the TCGA database and found that the expression of Quaking (QKI), ADARB2 and muscleblind-like splicing regulator 1 (MBNL1) was significantly decreased in CRC tissues compared with paired normal tissues, which was consistent with the circRHOBTB3 level (Figure S5C). To examine whether these SFs are involved in circRHOBTB3 generation, we designed 2 siRNAs that specifically targeted QKI, ADARB2 and MBNL1 and then assessed the knockdown efficiency by qRT-PCR (Figure S5D-E). The expression of circRHOBTB3 was further evaluated in SW480 cells after transfection of the siRNAs of these SFs, and the results indicated that only ADARB2 knockdown resulted in a reduction in circRHOBTB3 expression (Figure 7B and Figure S5E). The IHC results confirmed that the protein expression level of ADARB2 was lower in CRC tissues than in adjacent normal tissues (Figure 7C). We also measured the level of ADARB2 in tumor tissues from 40 CRC patients and found that it was positively correlated with the level of circRHOBTB3 (Figure 7D). Therefore, we hypothesized that both FUS and ADARB2 may be involved in generating circRHOBTB3 in CRC cell lines. qRT-PCR showed that silencing FUS and ADARB2 resulted in a more significant decrease in circRHOBTB3 expression in DLD-1 and SW480 cells but had no effects on the linear isoform and precursor mRNA (pre-mRNA) of RHOBTB3 (Figure 7E).

As reported previously, many SFs could contribute to the production of circRNAs by binding to specific motifs in the flanking introns within 500 nt proximal to back-splicing sites 42, 43. By sequence scanning, we identified canonical FUS binding motifs (GGUG, GUGGU, or GGU) inserted in both upstream and downstream introns flanking the circularized exons of circRHOBTB3 44-46. Moreover, base pairing between inverted repeats, such as Alu elements necessary for ADARB2 binding, has been found both upstream and downstream of circRHOBTB3 (Word S1). By using NCBI BLAST, the upstream and downstream AluY elements were found to be in an inverted orientation and highly reverse-complementary (89% identity; Word S1). Therefore, we designed 4 primers (a-d) targeting both upstream and downstream flanking sequences containing FUS binding motifs and 1 primer (e) targeting the downstream AluY element (Figure 7F). RIP analysis further confirmed the binding between FUS and flanking introns as well as the binding between ADARB2 and Alu elements (Figure 7G-H). Together, these results identified FUS and ADARB2 as vital regulators of circRHOBTB3 production in CRC cells by binding to specific motifs and Alu elements in the introns flanking the circRHOBTB3-forming exons.

## Discussion

CircRNAs can be detected in various tissues, exosomes and body fluids, including blood, urine, cerebrospinal fluid and saliva 47. Due to their abundance and high stability, circRNAs are suitable as biomarkers for diagnosing and monitoring diseases and establishing prognoses 8, 48. In this study, we observed significant downregulation of circRHOBTB3 in CRC tissues, especially in advanced clinical stages (stage III and IV). Moreover, the analysis of clinicopathological characteristics indicated that circRHOBTB3 levels are negatively correlated with the tumor size and lymph node and nerve metastasis. These data strongly suggested that circRHOBTB3 may serve as a valuable predictive tissue biomarker in CRC progression. ROC analysis indicated the diagnostic potential of circRHOBTB3 in CRC patients with different clinical characteristics. However, the limitation is that the number of patients included was limited and the time of inclusion was not long enough. We will continue to expand the number of patients and follow up with the patients to obtain survival information and assess the ability of circRHOBTB3 as a prognostic marker in CRC. Furthermore, the levels of serum circRHOBTB3 expression could be analyzed in further studies to assess its ability as a liquid biopsy biomarker.

The miRNA sponge function of circRNAs has been questioned partially because their abundance is far less than that of miRNAs, and the majority of circRNAs harbor far fewer miRNA binding sites than ciRS-7 and circZNF91, preventing them from achieving the miRNA sponge function 12, 49. However, the patterns of circRNA-protein interactions are more complex and interesting than those of circRNA-miRNA interactions and have been the subject of great focus recently 50. RBPs affect all phases of the circRNA lifecycle, including circRNA biogenesis, localization, function and degradation 51. Herein, we determined that increased expression of circRHOBTB3 restrains PTBP1 expression by binding to HuR and facilitating its ubiquitination and degradation, which consequently restrains CRC metastasis. Moreover, FUS and ADARB2 are responsible for the generation of circRHOBTB3 in CRC cells.

As a universally expressed and evolutionarily conserved RBP, HuR exerts oncogenic effects mainly by stabilizing cancer-related mRNAs with adenylate uridylate (AU)-rich regions in 3'UTRs in the cytoplasm 52. During CRC initiation and progression, HuR is significantly increased in the cytoplasm through the nucleocytoplasmic shuttling element and phosphorylation mediated by several cancer-associated kinases 53, 54. In the present study, we found the interaction between circRHOBTB3 and HuR by performing bioinformatics analysis, and upregulation of circRHOBTB3 renders HuR susceptible to ubiquitination via the specific E3 ubiquitin ligase β-Trcp1. Since the pulldown experiment did not detect the binding of β-Trcp1 to circRHOBTB3, we proposed that the interaction of circRHOBTB3 with HuR could make it more accessible to β-Trcp1. The specific mechanism needs further exploration. In addition, we cannot exclude other mechanisms because circRHOBTB3 has been reported as an miRNA sponge in gastric cancer (GC), and only a fraction of circRHOBTB3-repressed genes are also known HuR targets according to our RNA-seq analysis 55.

Recently, an increasing number of studies have shown that circRNAs can interact with regulatory RBPs and further affect the fate of their target mRNAs 56. PTBP1 (also termed heterogeneous nuclear ribonucleoprotein I, hnRNPI) is a multifunctional RBP and an SF involved in various biological processes 57. The oncogenic role of PTBP1 has been detected in different cancer types, including CRC, and high PTBP1 levels are associated with accelerated invasiveness and poor prognoses 38, 58. Moreover, PTBP1 modulates the alternative splicing of PKM and the generation of PKM2, which is ubiquitously increased in CRC progression to promote the Warburg effect 39, 59. Our study demonstrated that circRHOBTB3 binds to HuR to reduce its expression, resulting in a decrease in the control of PTBP1 mRNA stability. However, phenotyping indicated that the effects of circRHOBTB3 on CRC cell metastasis cannot be completely reversed by PTBP1, suggesting that circRHOBTB3 may have additional mechanisms that need to be further explored.

To gain a deeper understanding of cell type-specific expression and the role of circRHOBTB3, we further investigated its biogenesis mediated by pre-mRNA splicing. First, research has shown that MBL protein and the combination of numerous hnRNPs and SR proteins could regulate RNA circularization in Drosophila 60. In addition, some RBPs, such as QKI and FUS, could bind to specific motifs in the flanking introns of circularized exons and dimerize to form a looped structure 61, while adenosine deaminase enzymes (ADARs) and ATP-dependent RNA helicase A (DHX9) prevent circularization by disrupting base pairing between inverted repeated Alu (IRAlu) elements 42, 62. Interestingly, the RNA-editing enzyme ADARB2 was reported to act as a suppressor of ADAR family members and enabled the formation of circNT5E in glioblastoma (GBM) 62. Since linear splicing and back-splicing utilize the same pre-mRNA, they possibly compete with each other 41. Some studies on circRNA generation indicated that inhibition of circRNA production seems to increase the linear mRNA levels of host genes 62, 63, while other studies did not mention the variation in the linear counterparts 64, 65. Our study confirmed that the combination of FUS and ADADB2 promotes endogenous circularization signaling in circRHOBTB3 biogenesis, supporting the idea that SFs can contribute to cell type-specific circRNA formation. However, the decrease in circRHOBTB3 expression seems to have no effects on the linRHOBTB3 promotion, which is similar to findings by Yu J et al. and Wu Y et al. 66, 67. We cannot rule out the possibility that cricRHOBTB3 biogenesis is regulated by other SFs, and further understanding of pre-mRNA splicing may elucidate how circularization-related SFs function to regulate the circular/linear ratio.

Current functional and mechanistic insights highlight the vital role of circRHOBTB3 in CRC metastasis. However, whether circRHOBTB3 can be utilized in clinical practice as a potential therapeutic target still needs to be addressed in further studies. For tumor suppressive circRNAs, the induction of their expression in specific cancer types may promote antitumor effects 68. For this purpose, delivering engineered circRNAs by specific vectors designed for circRNA expression or transfecting purified, *in vitro*-generated circRNAs might help downregulated functional circRNAs become promising therapeutic targets. Moreover, we are pleased to find that researchers have constructed artificial circRNAs using *in vitro* enzymatic ligation. These synthetic circRNAs can be stably expressed in cancer cells and act as miRNA sponges (miR-21 and miR-93) or protein sponges (hnRNPL) to counteract miRNA or protein functions 69-71. Therefore, the artificial circRNAs could be novel tools for anticancer research and future molecular therapy.

## Conclusion

In summary, our study is the first to identify circRHOBTB3 as a novel tumor suppressor in CRC aggressiveness. CircRHOBTB3 exerts its effects by destabilizing HuR to regulate the levels of PTBP1-induced genes involved in cancer metastasis. These observations support the idea that circRNAs may modulate the expression of a wide range of cancer-related genes at the posttranscriptional level by interacting with RBPs and regulating protein ubiquitination.

## Supplementary Material

Supplementary figures and tables.

## Figures and Tables

**Table 1:** Relationship between circRHOBTB3 expression level and clinicopathological features of CRC patients

Clinicopathological parameters	N = 83	circRHOBTB3 expression		P value
		Low expression (n = 42)	High expression (n = 41)	
**Gender**				
Male	52	25	27	
Female	31	17	14	0.551
**Age**				
< 60	27	10	17	
≥ 60	56	32	24	0.086
**Tumor location**				
Colon	42	22	20	
Rectum	41	20	21	0.743
**Differentiation**				
Well and Moderate	59	31	28	
Poor	17	7	10	0.833
**Clinical stage**				
I + II	39	14	25	
III + IV	44	28	16	**0.012**
**T stage**				
T1-2	16	4	12	
T3-4	67	38	29	**0.023**
**Lymph node metastasis**				
Absent	43	15	28	
Present	40	27	13	**0.003**
**Distant metastasis**				
Absent	70	33	37	
Present	13	9	4	0.144
**Nerve Invasion**				
Absent	72	33	39	
Present	10	9	1	**0.009**
**Tumor size**				
≤ 5 cm	65	29	36	
> 5 cm	18	13	5	**0.038**
**CEA level**				
< 5 µg/mL	49	26	23	
≥ 5 µg/mL	34	16	18	0.591
**Chronic diarrhea or constipation**				
Absent	52	25	27	
Present	31	17	14	0.551

CEA: carcinoembryonic antigen; P values were calculated using chi-square tests.

## Abbreviations

CRC: colorectal cancer
RHOBTB3: Rho-related BTB domain-containing protein 3
circRNA: circular RNA
siRNA: small interfering RNA
qRT-PCR: quantitative real-time polymerase chain reaction
FISH: fluorescence *in situ* hybridization
RIP: RNA immunoprecipitation
RBP: RNA-binding proteins
HuR (ELAVL1): human antigen R (ELAV like RNA binding protein 1)
PTBP1: polypyrimidine tract-binding protein 1
FUS: Fused in sarcoma
ADARB2: adenosine deaminase, RNA-specific B2
UBS: ubiquitination-proteasome system
EMT: epithelial-to-mesenchymal transition
PKM: pyruvate kinase
SF: splicing factor
