# Supplementary material for: Circular RNA circRHOBTB3 represses metastasis by regulating the HuR-mediated mRNA stability of PTBP1 in colorectal cancer
Source: Theranostics. 2021 Jun 1;11(15):7507–26. doi: 10.7150/thno.59546 (PMC8210600; doi:10.7150/thno.59546)
Supplement: Supplementary file 1 — Supplementary figures and tables. [file thnov11p7507s1.pdf]

Supplementary materials:

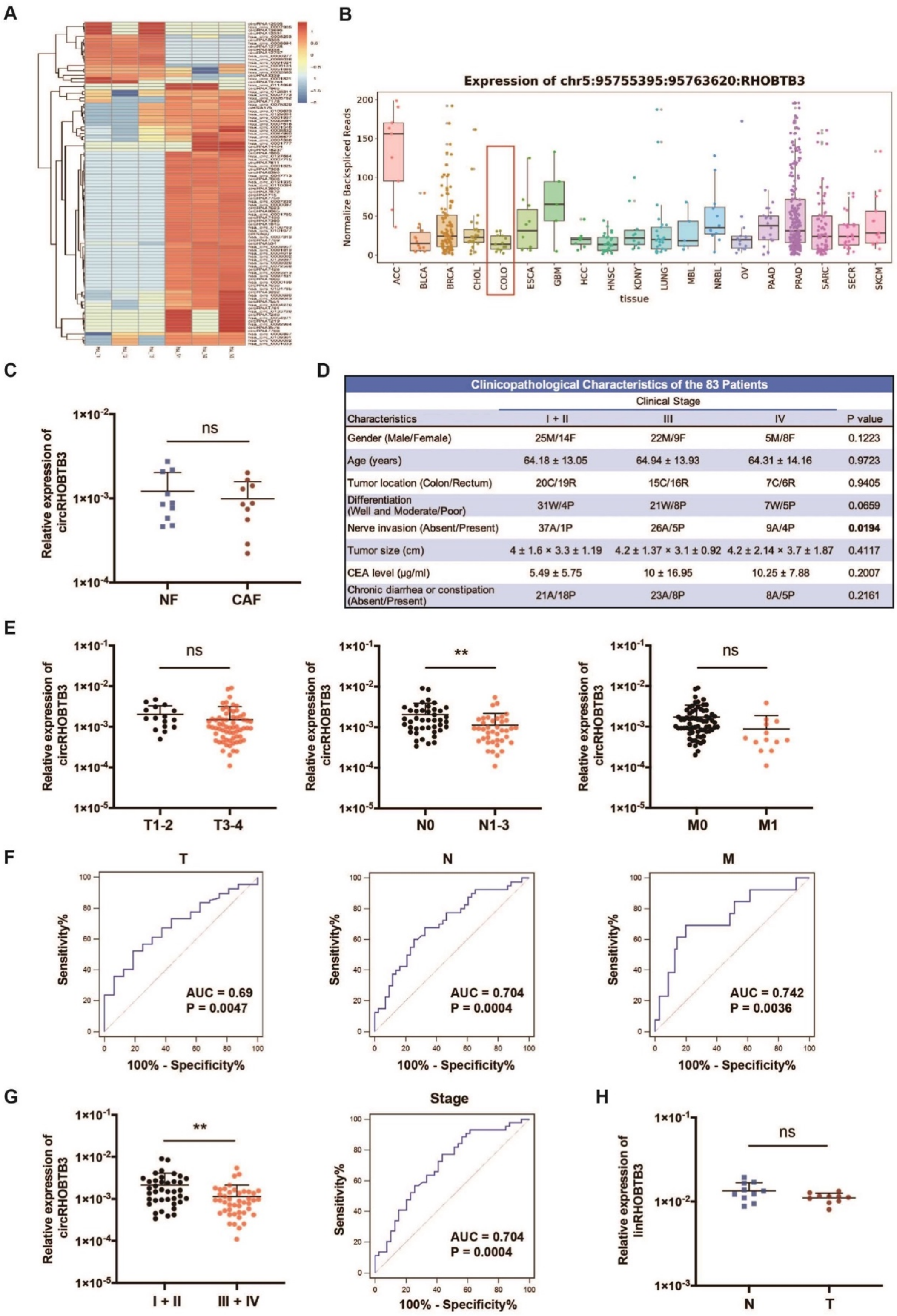

Figure S1. Validation and expression of circRHOBTB3 and linRHOBTB3 in

different tissues. (A) Heatmap of differentially expressed circRNAs in paired CRC tissues and normal tissues (n = 6). **(B)** The overall expression of circRHOBTB3 in multiple human cancers from the MiOncoCirc database. **(C)** qRT-PCR showed the circRHOBTB3 levels in primary cultured CAFs and paired NFs from 10 CRC patients. The P values were determined by paired Student's t-tests. **(D)** Clinicopathological characteristics of the 83 CRC patients. **(E)** qRT-PCR showed the circRHOBTB3 levels in tumor tissues from 83 CRC patients with different clinical characteristics. **(F)** ROC analysis showed the diagnostic value of circRHOBTB3 in CRC patients with different clinical characteristics. **(G)** qRT-PCR and ROC analysis showed the levels and diagnostic value of circRHOBTB3 in 83 CRC patients at different clinical stages. **(H)** qRT-PCR showed the relative expression of linRHOBTB3 in CRC and adjacent normal mucosa tissues (n = 10). The P values were determined by paired Student's t-tests. The data are presented as the mean ± SD of three independent experiments, two-tailed Student's t-tests, $*p < 0.05$, $**p < 0.01$ and $***p < 0.001$.

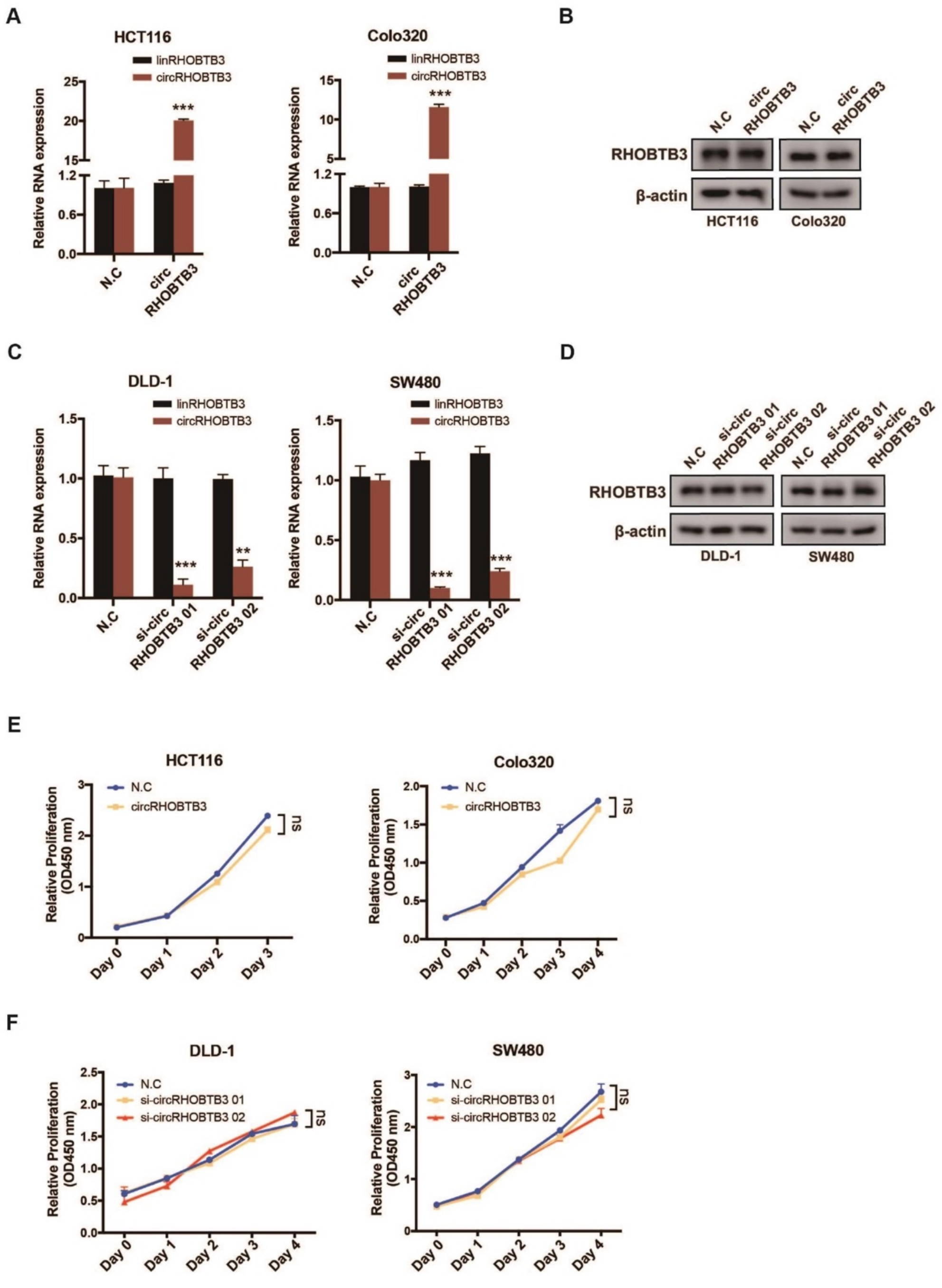

Figure S2. Transfection efficiency and regulation of CRC cell proliferation of circRHOBTB3. (A) and (B) qRT-PCR and Western blotting detected the expression of circRHOBTB3, linRHOBTB3 and RHOBTB3 protein in HCT116 and Colo320 cells after stable transfection of the N.C or circRHOBTB3 plasmid. **(C) and (D)** qRT-

PCR and Western blotting detected the expression of circRHOBTB3, linRHOBTB3 and RHOBTB3 protein in DLD-1 and SW480 cells after transfection of circRHOBTB3 N.C (control siRNA) or siRNAs. **(E)** CCK-8 assay assessed the proliferation of HCT116 and Colo320 cells transfected with the N.C or circRHOBTB3 plasmid. **(F)** CCK-8 assay assessed the proliferation of DLD-1 and SW480 cells transfected with N.C or circRHOBTB3 siRNAs. The P values in **E** and **F** were determined by two-way ANOVA. The data are presented as the mean ± SD of three independent experiments, two-tailed Student's t-tests, *$p < 0.05$, **$p < 0.01$ and ***$p < 0.001$.

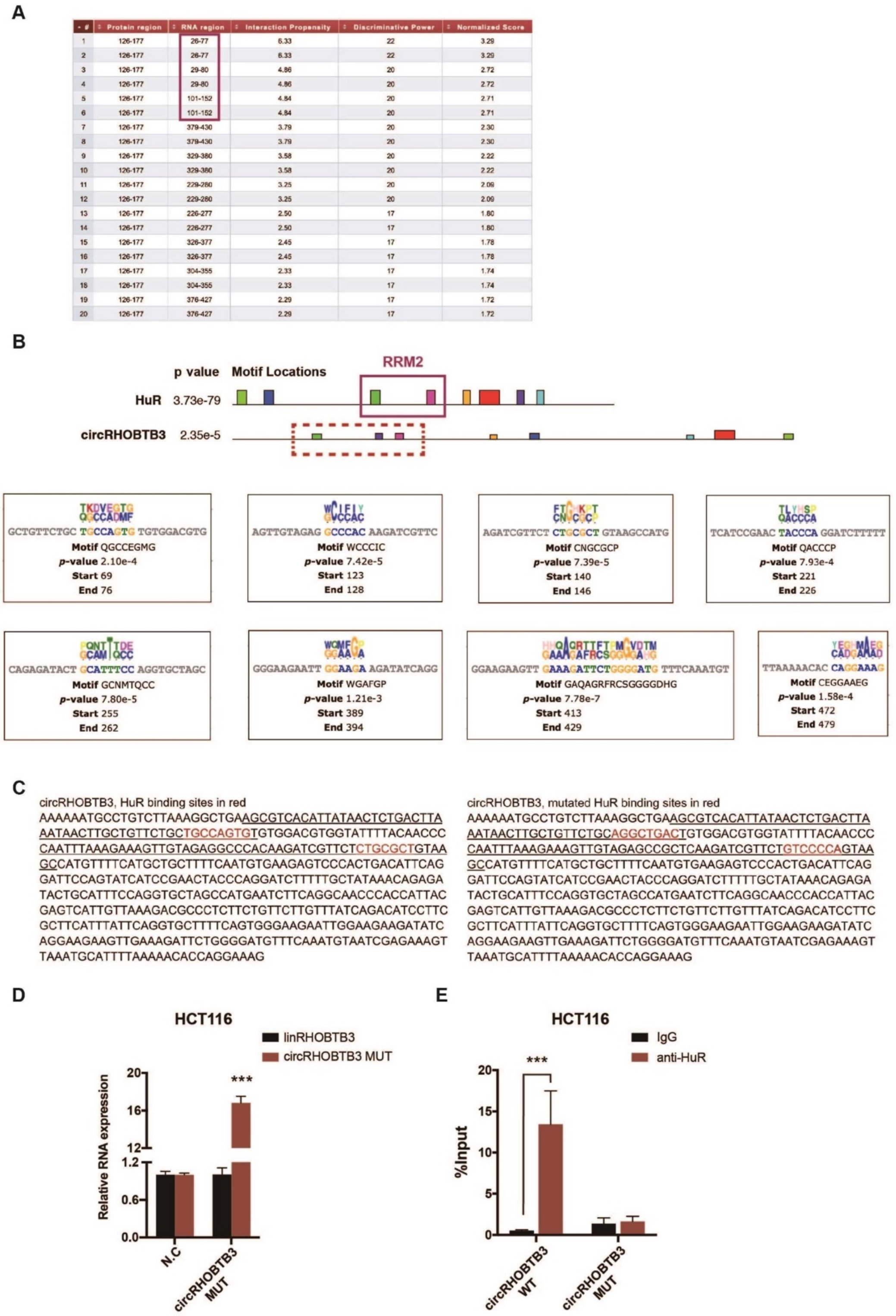

#	Protein region	RNA region	Interaction Propensity	Discriminative Power	Normalized Score
1	126-177	26-77	6.33	22	3.29
2	126-177	26-77	6.33	22	3.29
3	126-177	29-80	4.86	20	2.72
4	126-177	29-80	4.86	20	2.72
5	126-177	101-152	4.84	20	2.71
6	126-177	101-152	4.84	20	2.71
7	126-177	379-430	3.79	20	2.30
8	126-177	379-430	3.79	20	2.30
9	126-177	329-380	3.58	20	2.22
10	126-177	329-380	3.58	20	2.22
11	126-177	229-280	3.25	20	2.09
12	126-177	229-280	3.25	20	2.09
13	126-177	226-277	2.50	17	1.80
14	126-177	226-277	2.50	17	1.80
15	126-177	326-377	2.45	17	1.78
16	126-177	326-377	2.45	17	1.78
17	126-177	304-355	2.33	17	1.74
18	126-177	304-355	2.33	17	1.74
19	126-177	376-427	2.29	17	1.72
20	126-177	376-427	2.29	17	1.72

Figure S3. Identification of the motifs in the circRHOBTB3 sequence that interact with HuR. (A) Prediction of the binding positions of circRHOBTB3 on the HuR protein from the catRAPID database. **(B)** Prediction of the binding motifs of

circRHOBTB3 with the HuR protein from the MEME database. **(C)** circRHOBTB3 sequence labeling HuR-binding sites (red) and mutated nucleotides (red). **(D)** qRT-PCR detected the expression of mutated circRHOBTB3 and linRHOBTB3 after transfection of the circRHOBTB3 MUT or N.C plasmid. **(E)** RIP experiments confirmed the combination of HuR with WT circRHOBTB3 but not MUT circRHOBTB3. The data are presented as the mean ± SD of three independent experiments, two-tailed Student's t-tests, *$p < 0.05$, **$p < 0.01$ and ***$p < 0.001$.

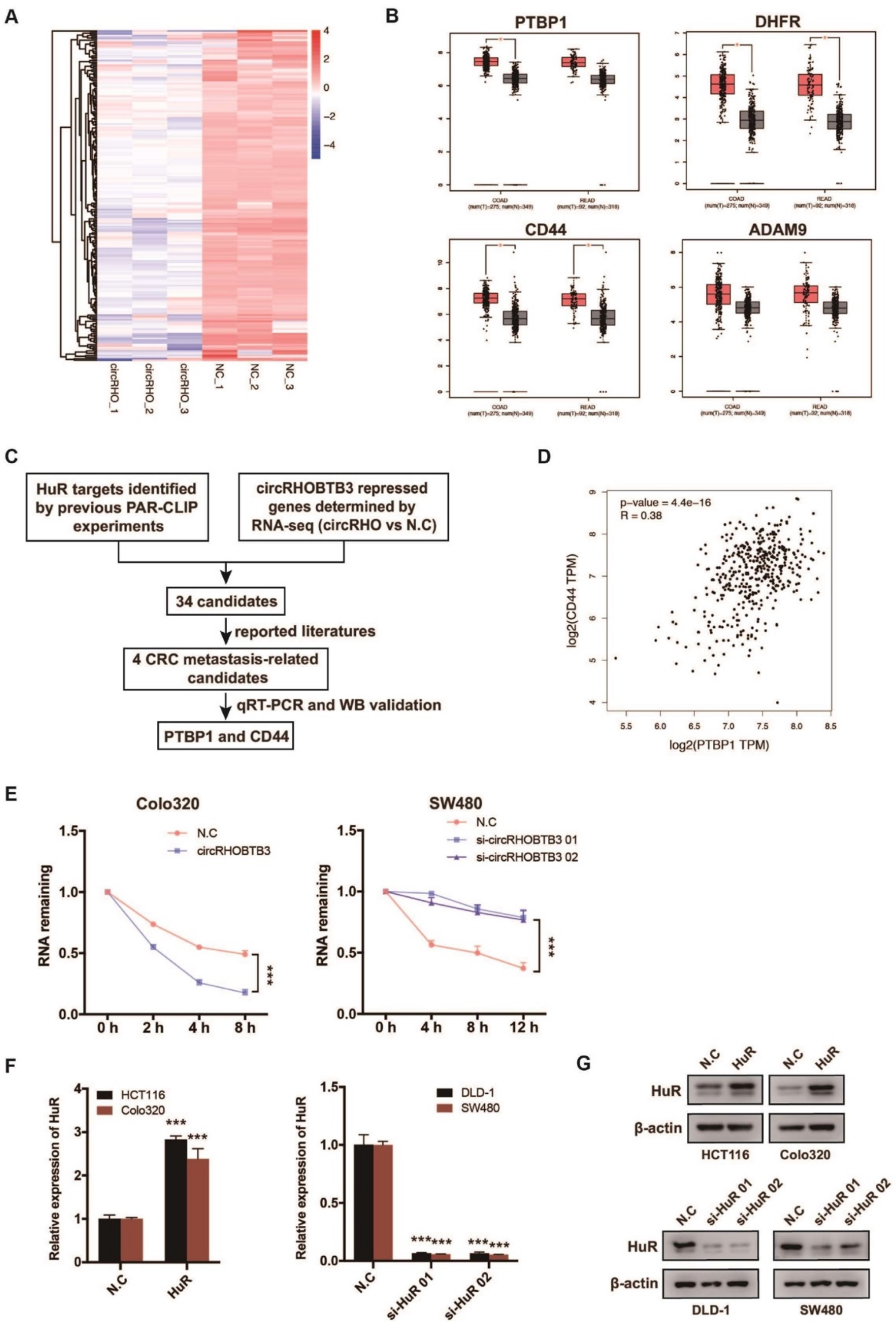

Figure S4. Identification of PTBP1 as the target of the circRHOBTB3/HuR axis.

(A) Clustered heatmap showing significantly downregulated mRNAs in circRHOBTB3-overexpressing HCT116 cells relative to N.C cells (n = 6, Log2FC >

1). **(B)** The expression of 4 selected metastasis-related genes in CRC tissues and adjacent normal tissues from TCGA (Log2FC > 1). **(C)** Flowchart illustrating the criteria for identifying PTBP1 and CD44 as targets of circRHOBTB3. **(D)** Positive correlation between PTBP1 and CD44 mRNA levels from TCGA. **(E)** qRT-PCR estimated the influences of circRHOBTB3 on the mRNA stability of PTBP1 in Colo320 and SW480 cells treated with actinomycin D. The P values were determined by two-way ANOVA. **(F) and (G)** qRT-PCR and Western blotting detected the expression of HuR mRNA and protein levels in CRC cells after transfection of HuR plasmid or siRNAs compared with N.C groups. The data are presented as the mean ± SD of three independent experiments, two-tailed Student's t-tests, *$p < 0.05$, **$p < 0.01$ and ***$p < 0.001$.

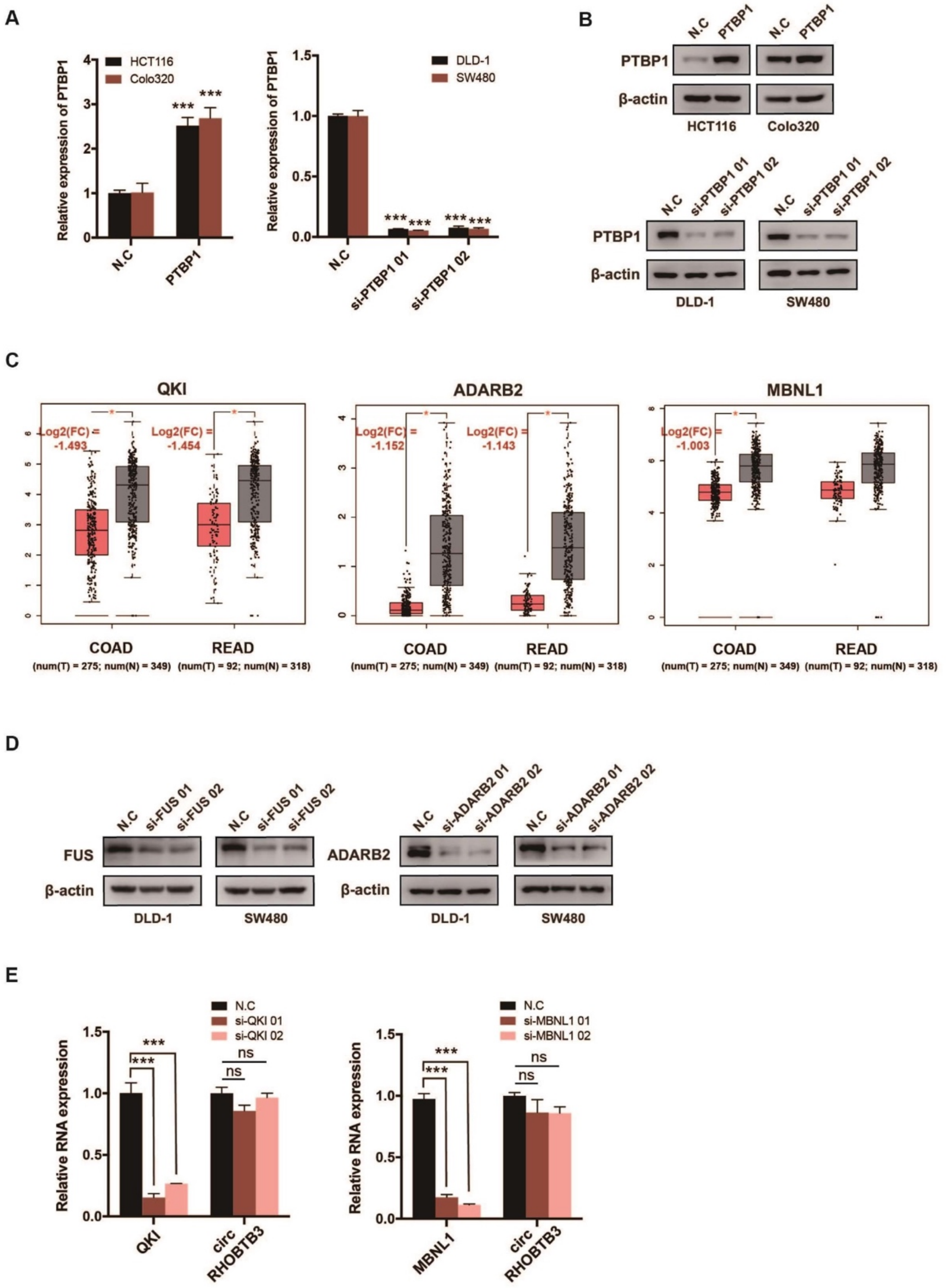

Figure S5. Downregulated RNA-editing RBPs in CRC tissues and their impact on circRHOBTB3 formation. (A) and (B) qRT-PCR and Western blotting detected the mRNA and protein expression levels of PTBP1 in CRC cells after transfection of PTBP1 plasmid or siRNAs compared with the N.C groups. **(C)** Significantly

downregulated RNA-editing enzymes in CRC tissues and adjacent normal tissues from TCGA (Log2FC > 1). **(D)** Western blotting detected the protein expression of FUS and ADARB2 in DLD-1 and SW480 cells after transfection with N.C or siRNAs. **(E)** qRT-PCR analysis of the expression of circRHOBTB3 upon QKI and MBNL1 depletion using siRNAs in SW480 cells. The data are presented as the mean ± SD of three independent experiments, two-tailed Student's t-tests, *$p < 0.05$, **$p < 0.01$ and ***$p < 0.001$.

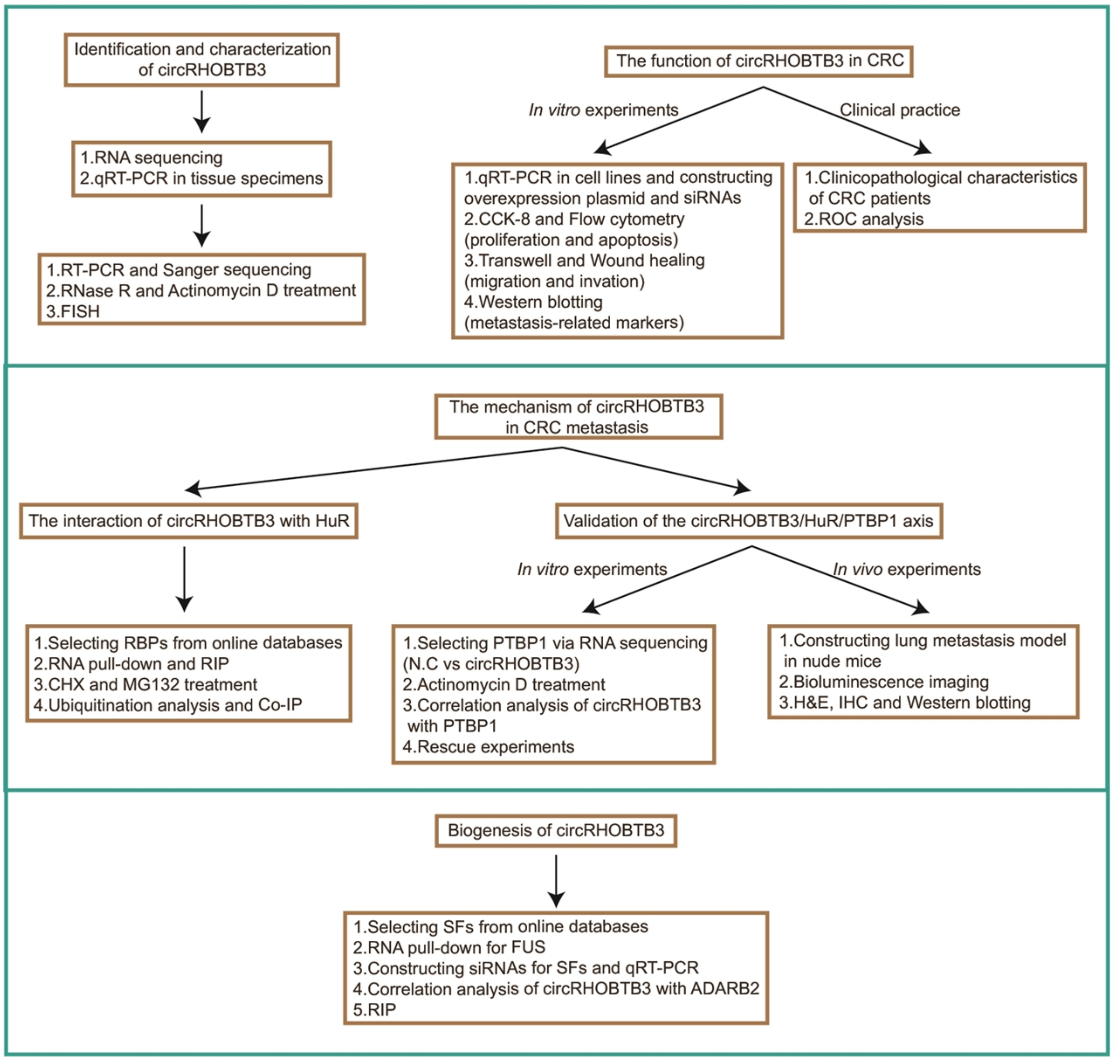

Figure S6. Flowchart illustrating our experimental procedure.

Table S1. The sequences of all primers and oligonucleotides used in the study.

Gene	Forward primer (5' to 3')	Reverse primer (5' to 3')
circ_0006867	TCCAGTGTTTCCTTGGGTCA	TTCCATGCAGCCCTTCTGTA
circ_0000690	CGCGAGCGAGTTTGTGAAAA	GGAACACCTCTGTCCCTCAA
circ_0008832	TAATTTCACAGCCCCAGGTG	AGTCATGCTAGGAGAAGCTGTG
circ_0004524	GGCTTGCCACCAACAGAATAAA	AGTCTGTCTGATGGTTGACTGG
circ_0001546	TGTCATCGAGAAGGCAGAGA	GAATTCTTGGCTGGCTCACA
circ_0007444	TTCAGTGGGAAGAATTGGAAGAA	TGGCAGCAGAACAGCAAGTTA
circ_0004368	CACCAGCAGAAGAACCAAATATGA	TTCTCATATTTGGTTCTTCTGCTGG
circ_0009043	AGCATCCGCAAACATTCAGAC	TGCTTCAGCTCTTCCATTGCTA
circ_0002490	AGCTTGTGTTCAGGTGTGTCA	ATACACTTGGTGGGATCTGCT
circ_0001535	GAAGAAAATACCCAGCACCCA	TCAAAAAGGTGCTGTTCCACA
linRHOBTB3	GCCCTTGGATTTGAGATCATGT	GTCAGCGCCCCCAATTAGAT
β-actin	AGAGCTACGAGCTGCCTGAC	AGCACTGTGTTGGCGTACAG
HuR	CAGCATTGGTGAAGTTGAATCTG	CCTTAATGGTTTTTGACTGGAGC
PTBP1	ATGGACGGCATTGTCCCAGATATAG	CTAGATGGTGGACTTGGAGAAGGAGA
DHFR	TTCCAGAGAATGACCACAACCT	TGCCACCAACTATCCAGACC
CD44	CTGCCGCTTTGCAGGTGTA	CATTGTGGGCAAGGTGCTATT
ADAM9	TCTTGCCACAGACCCGGTAT	ATCTCCAGTCCAACTAGCACA
FUS	TTATGGCCAGAGCCAGAACAC	GCTACCGTAACTTCCCGAGG
QKI	AAGCCCACCCCAGATTACCT	ACTCTGCTAATTTCTTCGTCCAG
ADARB2	CCTAAACACCAAGGCGAGAT	AAAACTAACGGGGCACACTG
MBNL1	GCTGTTAGTGTCACACCAATTCG	AGGCGATTACTCGTCCATTTTC
pre-RHOBTB3	CTGCTGTGAGTAGGAAGGACA	ACTTTCCCTTATCACACCATATCC
circRHOBTB3 MUT	TTCAGTGGGAAGAATTGGAAGAA	AGCCTGCAGAACAGCAAGTTA
Oligonucleotide	Sense (5' to 3')	Antisense (5' to 3')
si-circRHOBTB3 01	AACACCAGGAAAGAAAAAA	
si-circRHOBTB3 02	AAAGAAAAAATGCCTGTCT	
si-HuR 01	GGUUUGGGCGGAUCAUCAATT	UUGAUGAUCCGCCCAAACCTT
si-HuR 02	GAACGAAUUUGAUCGUCAATT	UUGACGAUCAAAUUCGUUCTT
si-PTBP1 01	CCCUCAUUGACCUGCACAATT	UUGUGCAGGUCAAUGAGGGTT
si-PTBP1 02	GCCUCAACGUCAAGUACAATT	UUGUACUUGACGUUGAGGCTT
si-FUS 01	CGUGGUGGCUUCAAUAAAUTT	AUUUAUUGAAGCCACCACGTT
si-FUS 02	GAGCAGCUAUUCUUCUUAUTT	AUAAGAAGAAUAGCUGCUCTT
si-QKI 01	GCUGCUCCAAGGAUCAUUATT	UAAUGAUCCUUGGAGCAGCTT
si-QKI 02	GGAGCAUCUAAAUGAAGAUTT	AUCUUCAUUUAGAUGCUCCTT
si-ADARB2 01	GCUGAGCAGUCAACUCAAATT	UUUGAGUUGACUGCUCAGCTT
si-ADARB2 02	CGGACCUGGAGAUUAUCAATT	UUGAUAAUCUCCAGGUCCGTT
si-MBNL1 01	CCAGACACGGAAUGUAAAUTT	AUUUACAUUCCGUGUCUGGTT
si-MBNL1 02	GCCUGGUCCCGGCAGAGAUTT	AUCUCUGCCGGGACCAGGCTT

Word S1. The sequence of Alu elements of circRHOBTB3 analysis

>hg19_hub_77_jeck_circRNAs range = chr5:95086100-95104324 5'pad = 5000 3'pad = 5000 strand = + repeatMasking = none
TATGGCAACCACTAGCTACATGTGGCTATTTATATTTAAATGAATTGAAAT
AAAACCAAGTAAAATTCAGTTCTTCAAGCATATGAACCATATTTTAGGTG
CTCTATGGCTACATGTGGCTAGTGGCTACTGTATTATATAATGCATATATT
ACAGAACATTTCCATCAACACAGAAAGGTCTATGGAATAGTGCTAGGACT
ATGCTCCCAAATCCAGTACCCCAGAATTCTCCGGCCACTGGTCCTGGGTCT
GTTTCGGCCTGTGCAGGCCATTTTCCTGGGACAGTCCTCCTGATGGTGGAC
CACATTGCTGGTATATGCACCCCTAGGCCTGCGGGGTGGCCAAAGGGTGA
GTCTTTATGGGGAATGGGGGAGGGGACAGTGGACAGAGCTTTGATGTCCA
CTTGTGTGCACAGAGCTCTTTAAAGCTCTGTGTTAGAGCAGGAGGTGAGA
AGAGAATGGGTGGTCTTAGGGCTCAGGAATTAGGGATCAGCTCTCCCTTT
GCTGTATGAACCTCTGAAGAATCTGAGAATTTAAATATAAATCTTGCCTGC
CAGGCTTTTATGATGATGTATTTGTCAAAACAGAAGTATAGAATATCTTTT
ATATAACAGTTTGTTTTCTTGATTTACAGCGTTTAAATATTTAGGTGTGTG
GTGGGTGGGCCTCCATTTGGACTTTTGCCCCAGGCCTTGCAAGCTAGGGC
ACAGGCCTGAAGCTACGTAAATGCACCTGAGAGAGATTTTTAGTTTGTAA
AGGACAGATTTTAAAGAAATTTGTTCAAGAGAAGAAAGCGAAGTTTCCTG
GCTCATGGGAACATCTGTAGACATAGAGGCAGCTCTGTTCCTGCTTTAATT
TCACTAATATTAACTGTGGTGGTTGCCCACTGAGAAAATTATTACCAGCCA
CACAATTGTAGTGCTTGGCTTTCTCGAGTATTCAAGAGAGTAGCTGCGTGG
GATGGCATGCACTTATAATCCCAGTGACTTGGAAGGCTGAGGCGGGAGGA
TCCCTTGAGTCCAGGAGTTTGAGTCTAGCTTGAGCAACATAGTGATACTCC
ATTTCTAAAATTAAAAAAAAAAAAAAGAATATATATATATATTTGTTAAA
TTTGAAAAAGAGAACCAACAATTCCTTCCTTGAAAAAAAAACTTTTATTTT
AGGTTCAGGGGTACATATTTGTTATACGGGTAAATTGCATGTCACAGGGG
TTTAGTGTTCAGATTATTTCACTAACCAGGTAATAAACATAGTACATGGTA
GATAGTTTTCTGATCCTCACCCTCCTCCCTCCCACCACCCTCTCAAGTAGG
CTCTGGTGTCTGCCGGTCACTTCTTTGTGTCCATATGTACTCAATGTTTAGC
TCCCACTTATAAGTGAGAACATGTGGTATTTGGTTTCTTGTTCCTGTTAGTT
CGTTTAGGATAATGGCCTCTAGCTCCATCCATTTTGCCGCAAAGGACATAG
TTTCATTCTTTTTATGTCTGCATAGTATTCCATCGTGTATATGCGCCACGTT
TTCTTTATCCAGTCTATCGGTGATGGACATTTAAGTTGACTCTATGTCATT
GCTGTTGTGAATAGTGCATGTTTCTTTGTGGCAGAATGATTAATAACAATT
CCTTCTTGAGTGGATAGTTTATTTATGTTCAGAGAAGGCACAATCTGCTCC
ACTCGATTCATTTTTTAAATAAAAACATTGTTTATATGTGTTTGCTAATGC
ATACGTATAAATGGTACTGACGTTTAAAATGTTTGACAATTGTGACTTCAG
ATGCAAATTTTCATGTTGGATATATAGTTTTGTTGGATCTTCAATTAAAAT
TTGATTCCTGTTTTAGTTGGAGTATTTTATGATTCAAGCCTTAAATCAGAA
GACAAGTGAAAAAATGAAGAAAAGAAAAATGAGCAACTCCTTTCATGGA
ATTAGACCACCTCAACTTGAACAACCAGGTGCATTTCTTAGCCAATGTCTA
GACATTCATTAAACATTCATTACAGATCCTTTCTCACAGGTCTTAGAGCAA

TTATTCTCAAACTTGAGCATGCTCAGAGCTTACCTGACATTCTGATTCAGT
CAACCTGGGATAAGGCCTAGAATCTGTGTTAATCAGCCTCCCAAGTGATG
CAAAAGCAGATTGGTACTTGAATCACATTTTGAGAAATACTGCTTTAAAA
ATTGAGCCTATAGATCCTATGTGTCCTAGGTGGGGAGCTGTTCTAGATTAA
AGTCTAATGCTAAGTTACTACTAAATGCAATGCATGAACCTTGATTGGAT
ACTGGTTCAATAAAAACATCTAAAAGATGTTCTTGGGACATGGGTTAAAT
TTTAATACGGACTGGAAATTAGATTATATCATGTAATTACTGTTAATTTTC
TTATGCATCATAATGGTATTGTGATGAATAGGAGAATGTTCCTGTTCCCAG
GAGATGAAGGTGAACGTAGAGTCCCGGGCGCGGTGGCTCACACCTGTAAT
CCCAGCACTTTGGGAGGCCGAGGTGGGAGGATCACAAGGTCAGGAGATC
GAGACCATCCTGGCTAACACGGTGAAACCCCGTCTCCACTAAAAATACAA
AAAAATTAGCCGGGTGAGGTGGCAGGCGCCTGTAGTCCCAGCTACTCGGG
AGGCTGAGGCAGGAGAATGACGTGAACCCAGGAGGCGGAGCTTGCAGTG
AGCAGAGATTGTGCCACTGCACTGCACTCTGGCCTGGGCGACAGAGCAAG
ACTCTGTCTCAAAAAAAAAAAAGAAAGTGTTTTAGAGCCAAAGGATCCTG
ATATCTGCAACTTTTAAATATTTCAGCTGTGTGTATGTGTGTGTGTTGATG
TGTGGATGTGTGTGTGTGTGTGTGTGTGTGTGTGTGTGTCTTTCTGTCTCTC
TAGAGATAAGGGGAAAATAAAAAATGTTAATAATTGCAAAATCCACGTG
AGGGATATATGTGAAGGGTATATGTGTGTTCATTGCATCATTCTTTCAACT
TTTTTGACTGTTTGAAAATATTTTTAATACAAAGTTCAGGGCAAATAAACT
TTAAAATAAATAAATTATATCAACTGTTATCAGAATTTTCTGCATGATTTT
GTAATAGAAATAGTAGGATAGCACTTTGTTGGATCAATGTCCTTTGGAATT
TAAGGGACCTCATCTCTTTAAAAAAGTGCAGATATATAGACCACATATAT
ATATAAATGATACACACTTGGCTTTTAATTTCCTGGTGTGCAAAGTGAAAG
GGGAGACATGGTCTATTAGGTTGCAAACCCTTGGGCCTTAAATCCTTTCTG
GATTAGAAAATGGGTGGGAAATGACCACATAAATAATGTAGATAGTTAGT
TCTTAGTTCTTATTAATCAAGAAGACAAAATATACCAGATCCTCAGAGAA
AGCAAAACATTACATGTCATTTAGCTTTAAAAGAAATATCTTGGCCCAGG
TATGGTGGCTCATGCCTATAATTCCAGCACTTTGGGAGGCCAAGGTGGGC
AGATCACCAGGGGTCAGGAGTTCGAGACCAGTCTGGCCAACATGGTGAA
ACCCCGTTTCTACTAAAAATACAAAAATTAGCCGGGCATGATGGCGACGC
CTGTAGTCCCAGCTACTCGGAAGGCTGAGGCAGAGCTGCATGAACCAGGG
TGGCAGAGGTTGCAGTGAGCCAAGATCATACCACTGCACTCCAGTGAGAC
TCTGTCTCAAAAAAAAAGAAAAGAAAAGAAAAGAAAAATCTCTTGTGTG
GGTCTTCATGTGAGGGCACTAAGTGATATAGTGGACAGGGCTTTCAAAAG
TGTCTCTTCAGGAAATGCAGCCTCAGCGTTCACAAGGCTGTTTCTTGTACT
CTTCTGTTAAGAGAGCTGTGTGTATAACACTAAAGATATTCCAGAGGTTC
AGAATAATACAGTCTGGGTACATATTTGGGGCTGATTTGTTCCAAGGACG
GATCCTGGGCTCTGGGGCACATGGCATTTAGGGTTCTGTAAAGGGGCCAC
AGGCAGTAGAAGGGAGGAGGGTGGACTGGAATACAGTGCAGGGAGAGCC
TGGCCTCCAAAGGACTTCGTTATTATCTGTTTTATAGGCTGGGGTTTGCTT
TGGAATCAAGCATTTTTATTGCTAAAGAAGTTTGAATTCTACTGATTTAGA
CGGTTGCATACCCTTCAAACAACCCTTCACAAATGTCACTTTCCTCAGCTC
CTCTGGATAAAAATGAGATGGCAGATGTTTTACAGTTAGGGTCTGTGGTG

AGGGCCTGGAGTGTAGGTATTCTGAGTTGATTGAGGGCTCATCTCTCTTTG
TTGGTGATCAAACTAGTAAACTGAAGGTTATATTCTCTTTTAGAAATTAGG
GAGCTGAGTTAGCACTTTTGCTGAAGTGTTTACTAGAAGGAAAAAATGC
TAGTTTTGGAATCCAAATAAAGGGGTCACAATGTTTTCAAACGATCGAGT
TCAAGACTCTGAGCCTTGACTCTGAGAAGTTGCACAATTTTAACAGCTCA
GCCCTCTACCTCTACTTCTGGTCCCAGGAGGAAAAGGACTTCAGATGTGA
GATTGGGTTCTTTGGAACTTATTCAGTACTTTGAATTTTTAAAATCAAAGG
TAAAAGTAAAGAAGGATGACTGTGAAATACTAAAGTTCAGTATTTGGCGG
CTTGTTTTATTTTGGGACACACAAGTAAGAAGAAATGTTATTTTATTGTTC
CGTACGTCATTAGTGGGTTTTTCATTTGCTCTATAAAATGTAAATAAACAT
AGTATTTGTGTGTCTGTGTTATAACATTCTACAGTTCCTGAATAATTAGTTT
ATATATGTTTATAAATTAGGTTAAGCAGTAGTAGAAAATCGCTGTTTTTGT
GCATCAAAAAATGGTTAAATATTGGCTGTTTCTTATGCTTTAACCTAATAA
TATTTTAAAATATTATACAATTATATTTAAAGTAAACATGAAATTGTTGGG
TCTGTAAACCTGAAAGGAGTTATTATTTTTATTTTCTTTTTCAAAACAGA
AAAAATGCCTGTCTTAAAGGCTGAAGCGTCACATTATAACTCTGACTTAA
ATAACTTGCTGTTCTGCTGCCAGTGTGTGGACGTGGTATTTTACAACCCCA
ATTTAAAGAAAGTTGTAGAGGCCCACAAGATCGTTCTCTGCGCTGTAAGC
CATGTTTTCATGCTGCTTTTCAATGTGAAGAGTCCCACTGACATTCAGGAT
TCCAGTATCATCCGAACTACCCAGGATCTTTTTGCTATAAACAGAGATACT
GCATTTCCAGGTGCTAGCCATGAATCTTCAGGCAACCCACCATTACGAGT
CATTGTTAAAGACGCCCTCTTCTGTTCTTGTTTATCAGACATCCTTCGCTTC
ATTTATTCAGGTATCTTGTCAAGTGGTTCTGGTTACTTACAATCTCATAAG
CTTTGGAAATGAAATGTGCCTGTGACACTCAAGGGTACTGGTTAGTTTTAC
AGTGGTCTTTACCTACCATCTGAGATTAAGATTATACATGAAATATTATTT
GCTTTATATATAAGAATTGGTCCAAGCTTTCTGTTTTCCTTACAAATGTAC
TTGCCTCATCTTTTCCCTATCAATTTCATTTTATTGTGGTAAAATACATAGA
ACATAAATTTTACCATCTTAACCATTTTTAAGAACACAGTTCAGTGGAATT
AAACACATTCATAATGTTGTGTAACTATCACCACCATCCGTGTCCATAATT
CTTTGCATCTTATAAAACTGAAACTCTATACCCATTAAACGATGATTCCCC
ATTCCGACCTCTTTCCAGCCCCTGACAGCCACCATTCTACTTTCTGTCTCTG
TGATTTTAACTCTCCTAAGTGCCTCATGTAAGTGGAATCATACAGTATTTG
TCATTTTGTGATTGATGTATTTTACTTAGCATAATGTCTTAAAGGTTCATCC
AAGTTGTGGCATGTATCAGGATTTTCTTCCTTTTAAGGTTGATAATATTCT
GTAGTACATATAGACCACATTTTGTTTACCCATTCATTCATCCATGGACGC
TTGGGTTGCTTCCACTGTTTTAGCTATTGTGAGTAATGCTGCTGTGAACAT
AGTGTACAAATATCTCTTTTGAGACTCTGCTTTTATTTTATTTTTTGCATAT
ATACACAGAAGTGGAATTGCTGGATCACATGGTAATTCTGTTTTTGATTTT
TTACTGCCATACTGTTTTCCACACTAGCTATACCACTTTATGTGCCTACAA
ACTGTGCACAAGAGTTCCAATTTCTCCACATCCTTGCCAACACTTGCTGTT
TATTTTTTTTAATAGTAGCCATCTTAATGGGTTTGATGTGGTATCTAATTCT
GGTATTGATTTGCATTTCCCTAATGATTAGTGATATCAAGCATCTTTTCAT
ATGTTTTTGTATTTGCCTTGGTTTTAAATCTACTTGTAAAAACCATAATCTG
CCAAACAAGTATGTTAATATTTTATTTCTTTAAAAAACATCCAAAATATGA

AATGTGAGAATTTATTGTCCAATTTAGTTTTAAAGACATAAAATATTCCAA
GAGTAAATGAAATCAAGTGTCTACACCTTAAAACCTAGCTATTCACTCTTC
GGTTTGACTCTTAGGTTTTGTAGGGCGTGAAAGAGAGAATTGCATTCAGC
TTACGGACTGGAAATCAATATCCAGTGTTAGTTGAGGTCTAATTAACTTTG
GCTATGACTATCATAGACGTTACTTTTTTTTTCCTTACTGTGCCCTTTCTT
TTTAAGATTTAGCTTATCAAGTATTTTTCTCTTTGCCCTTTTAAAAACTCAG
TAGCAACTATATTTTAAAGATGGAAGTTCTATCTGAGTCATTTTAGCAAAT
GAATTGAAGGTCTTACATCTAAGTGTCCCATGTAAGAGTGGAAAAGAACA
GATGTAAAATGAATTTTAATGTAAGCATCTAAATATAAGTTTTCTCAGAAC
TCCTGTCTTTAATTTTCTTTACCCACTTCAAATCATGTAGCGATAACCTGTT
AGGCTAAGGTCACTCCTGCTCTGCAGTGTGTCTGGAGAAACATTTATAATT
AATTTGTTTTTAGGGTTCAAGTAAAGCCTAATTGGGAACAGACAAAAAGT
TTAAGGTTTGCAAGGCAAGTATCATTTCATACAGGTAGGGAAATTTGGGG
CAGCCTTTATTTAAGTAATTGATGTTAAAAATAAGGTATTACTTTGTTCAT
GTGGTTTGTGGTCATGTAATTTTATAAGTTAAAACTTAAATTATGATTTTG
AAAAGTACAGTTGCAGCACGCAGATTTATGTGGATAAAAAATACATATTG
GAGTAGAATGTGGTGCTAGGTTAGTGAAATATTGGCTTGGAAAAAGCATA
AATTAAGCACCTGTACAGCACACACCCAGCACATTGCCTTCAACGGGTTC
TCTGTAAGTTTTAGTTGAATTAAAGAGTAGGCTCCAGTAGCCGGGCATCA
TCATATCAGTAAGCCAAACTATTTAAAGACTTCATTTAGTACTATAGATTT
TGACGTTTAAACTTAGAATGTCACAGTTCAGAATTTAAGATTTTGAAAAA
CTAACATGTTTACATGTTAAGAGAAAAAAGATGTTTTTAATCATATGTTTA
AGGAACTGTGAGGGATGTGACTTTTGTATGATAATTTTCTGTTGAAATTAC
ATGAATTATTCAGTAGGCTTCCACAATTATTATTGACTACTTTCAAAGGCA
TATGAGCACTCTGACGTCTTATATCCTTTTTAACATAGTAGCAGTAAAATA
GCTAGATGTATCTCCAGTGGCTGATCCTGCCTAGCTTTCTCCTAGGGGGAG
TGCTTTATTTAATTGCAAAATTAATACAGGCAGATGGAAAAAAAGAGAAA
GAAAAGAAAAAAGTTATCCTTCTACCCTAGACCCTTTCCCCGGAGTCATTT
ACCTGTTGTAATTTTTTTATGCACTCATTCTGATACTGTTTGAAAGAGATT
AATATTCTGGGGACATTTGATTAATAAAAGTGTAATCCTAGGTATCTGCTG
CTTTCCAGGGATCACAGGCCGTTTCCCATGGCTGGAGCGTAGAGTGAAAG
GTGATGGCTGGTGGGTGATGAGGTGGGCAAGGCCTGTGGAAGCTGTTCCT
GCTGCTCTTAGACTTGTGCTAGAGTTTTAAGCATGATCAGACTGGGCTTTA
GAAAGCTCATCTCTGGTTGTGGTTGCAGGGTGGAACAGTGCGGCCAAGAC
AGGCATTCAATCAGGAGGTCCCATTACTCTGGCGATCAGCCTCAGGAAGG
CCAACATTCAGACAGTGGGTGGCCCTGCCATTGGCCCTTCATCGTCTAGAC
CAGTGCTTTTCAAACCTTAAAGTGTGTGCAGATCATCTGAGGATCTTGTTC
CTGTGGAGATTCTGATTCAGTTACCTGGAGTGGGGCATGAGATTCTGCATT
TCCAGTAGTCTCCGAGGTGGTGCTGGATCTCTGGACTGCCAGAAGCAGCT
GGAATACCTGCTTTTGGAAGAACTGGGAACCAGCCAAAACCAGCAAACTA
GGGCCTAGAATTCAGTGCAGAGGCCCAGGAAGGCTACCAAGTTAACAGCT
TAGGATTCAAAGTAAAAATGGGGTGAGCTGTAAGGCTGACTGATGCAGTG
CTATTCAGAACTGGGGCTCTCAGTGCCCTGTGTGTCCCTGACGCGAGGAA
AAATGGATGCCTAGTGGTTGGGTGGAAATTAAGTAAGCCTGGAATAGGGA

TCGGAGGGCAGGCAGGCATGATGGTGTGGAGAGCCTGCTGAGCTACAGG
TCTTCAACCAGTTCAGAGTCCTGGCTTCTTAACCTGACACTGACCCGTCCC
ATTTCGGCTGTGATCTCTCCTTGTACGTCACGGCCAAAGCCGGAGGTGCTG
TCTGGTCTGAGCAGGGGCACGGGCCATTGACCCGGGGCTGCAGAGATCCA
GATGCTGGTTAAGGAAAAGGGAAAATAACTGGTTCTGCAGGTATCTATTT
ATTATTCTTTAATTATTTACAGATTACAACCTCTGGCTCAAGGAAAAGTTT
CATTTACATTATGTTAAAAAGAGATATCAAAGCTGTATTTGCACAGAAAA
GACAATTTTGAAAAATGCTGAAAATTAAGAGAAAAAAATTTACCCAGATT
CCACTATCAACAGAAAATGTTTAAACATTTATTGCTTTGTTTGATCCTCTA
GTCATTTGTTTGTGTATGAATAAGTGACACTGTTTTTGAAAAAATGTATTT
TGTAATCAAATCAAACAAAAATTTTAAACCTACGTTTTTCTCAGAGACTTC
AGTAAATGGGCCAGGCTTGTTTTGTTTTGTTTTTGTTTTAACATTTTTAAAC
CTGTGAATAGAATACAAGTGTTAAGGCACAGTTATGCAGTGGTTTTCCCA
CAGAAGTGCTGTGGTCTGCCCTTGGGTTATGCTAGAGAACCATTGAAGAC
AGGAGTTGTTTGTTTGTGTTCCCATCAGATGGGTTGGTAAAGCAGAGGTTG
AACTCCATCATGTGCTCAGGAATATTTGCCTTGAGCAAACACGGGCTAGA
GAGTTATAATAAGCCTATCTCCTGTCCTTGTTTTTTTTTTTTTTAAAGAACC
AGATTACAAAGAATATCTTAATTCTTTTTAAATAACATGCTTTTTTTAACC
TTTAGGAATTTGAAATTTGGTGTGTGTTATGTCACCATGAACATTTGGTTC
GTTATTTAGGAGCGACCTGTTTAACAACAGTCCGGTTAACATTTTACACCT
ATCTATTCCTATTCAAATTTTTGATTCAGTAGACACAGTCAAGGTATATCT
GTTTTGTATACGTTAGTTTTCTATTGAATAACAACAAACACTGCATAATGT
ATAAGTACTTAACTACTAATCCTAAGGAACGAGGAGTAATGTGATAAGTG
AAGCCAGCCCTTTCCTGGTCTTCAGGGAGTAATTCTTACATGGCATACAAT
TGTAACATTCTTATAATACTTGATACAGTGTTATGGTTGCACATTCTTGAG
TCATATGATCTCAGATGGTTAGGAAGTGAAGTATTTGTAAGAACCTCAAG
AATTTAGCTTTGAGCTCTTCCTGCCACAGATGGCCCTTAGAGTTTCACATC
ACTTATTGAAATGCCATCTGAGTAGACAAATATGTGGAAGCAAGCTTTCA
TCTTCAGTTTACATTCTCCAGTTCTTAGAATCATTCCGTGGCATGTCCTTTT
CTGTATTGCATAAAAACACAGCCTAGTATACATGAGTATACCATGCGCTC
TAAAAGTCTAAGTGGATTCAGTGCTTTAAGAAGTTAACTAAAGAAAATCC
CAGTCAGTGATGCAAGATGTCATCCTGCCCTTGTCTGCAGTTGATCAATCA
GTAAAGTTATGCAAGATGAGTAAGTTCTGGAGACCTGCTGTATGACATTG
TGCCTATTGTTAATGATATCGCATTATATACTTAAAGTTTTCTAAGAGGGT
AAATCTGATGTTAAATGTTTTTACCACTGGAAAAATAAAAGGAAAGTCCC
AGCCTTAGGATTACTGGTTTATTAGGACCTAAAAGTCTCAAAGATGACTT
GATTAATGTTTCTAATTATGAAAACAGCCCAATCAGCAGTGTGCTCTTGAG
AATTAGTCTTACTTTCATTTTCAGTGAGTGATTCTTCCTGTGTGTGTTCTCA
GACAGTTCAGTGGCCTGTGGGGATTTACCTGCTACTGATTCCATCTATTTA
GATTATTTGATGCTTGCTTGCTTTTAGATAATCTTCTGAATGGAGATATTTT
ATAGGTGTTTTTTAGTTAATATGCTTTTACTTAAATAACAATGTAACTTTTA
CATATATAAGTATGTAAAAGAACACAGAGACAGTACTTATGTTCTTCTTTA
GATTGGATGAGTGCAGACATAAAAAATACTTACATGTTCCCTTTCCTGCAT
ACCACTAACACTGGGTTTGGATAATGGCTGAAATTTCTTTCCTTTTTCCTCT

TTTTTTTTTTTTTTTTTTTTGAGACAGGGTCTCACTCTGTCACTCAGGCTGG
AGTGCAGTGGCATGATCATGGCTCACTGAAATCTCCGCCTCCTGGGTTCA
AGCGATTCTCCTGCCTCAGCCTCCCAGGTAGCTGGGATTACAGGCAGACG
CCACCATGCCCAGCTAATTTTTGTATTTTTAGTAGAGATAGGGTTTCCCCA
TGTTGGCCAGGCTGGTCTCGAACTCAGTGGTTGAAATTTCTAAAGGGAAA
GCAGTGGCCGTCAGCAGCATACCATGCACATTAGGGTCACCAGGGAGAAT
TTTAAAACACTGATGCCAGGGGCTTCAGGCCCAGAAATTCTAATTCACTT
GATCTGGAGATGAGACCTCCTCTCATGGTATTTTATAAAAGATCTCAGGTA
TTTATAATACACAGGTATGGTTGATAATCACTGGCATAGAGTAATTTTATT
GATCTGGGATCCATTGGCTTCTAAGGGGTTCTTACACTCTCACATTGTTAT
ACTGACTTTAATGTTTGTGTGCACATGAAATTTTCTGGTCATACAATCCCA
AGAATTTATGAGATGGGAAAAGAGGATGTGGCCACACAGTTAAAAAGCA
TTCATGCAGAAAGAGAAAAACAGAAATCATAGAGCTCTTCTACACTACAC
AGCTTTGAGAATTTGAGGAGGAAGGGGAGCAATCAGTGAAAGCATAAAA
AGAGCTAGCAAAGGCAGAAAGAAGCAGTGGTGTGCTGGAGCTGGCACCA
GCTTGTGAGAGCCCAGTTGTGTTTATCATCCCCATTTCCTTGTTCTGTGATG
TCATGTTGATAGCATCAAAAAATGGCAAATGCTACATATCATTTGACTCCC
TCCCCCAGGAGACACTGTAAGTATTAGGAAGGCCAGGTGGGGAGAAGTT
GAAAGGATATGCATTAGTGCTTAGTGCTGCAGGGCTCAGCTGTAAGAGCC
TGAGAGCTTCTGGACTTGCAATCTGCCCCTTCCAATTGGATTTGGTTTTAG
TTGGTACAGACTGGCTGCAATTGCCTGATCTCATTGGTTTAATAAGTGTTT
ATGTGCCTGCCTCATTCCCCAATATCCATGAAGGCATCTGGTGAGTGCTGA
ATACAGGAATGAAAACATCAGAAGAAATGAACTGAGAGCAGATAAAAAA
GGGTCTTGAATTACCCAGAGAGGTGAAGTGACTCATCACACTAGTAGTTA
ATAGCTGAGGTAGAACTGGATCCTAGGTCTCCTAACTATACCGCCCACAC
TAGGCTTCTTGAATTATATTTTTATGAGATAGGACCTTCTATAAATCATAG
GATGGATGCGTGTAATCTCTTGGAATGTCATGATCAGATTCGTGCTTTTGA
AAACTCACCAGCTTTGAGTAATAGGATGGATTTGATGGGGAAAAGGCTGT
TAGGAAGCAGGGAGGTTGGATAGGAAGTTGCTGCAGACCAAGTAAGAGA
AGTCCTTGGGGAAAAGGTCAGCTTTTGCTGACCATAGAATAAGAATTTTG
CAAGACATAGAGGAAAAGAGGCTGACAGAGTGGGAGAAGTGAAATGAGC
CTAGGAAGAGAGTTTTGAAGTGGGCTAGGGAAAGGCAAAGGACAGTGGG
GTGATTTTGCTCCGATGACATGAGCTACGTGGACGAGTAGCTCCCTTCCTC
AGGCTGGTTTTGTAGAGTTGGCTCTCAAGCATCCCAGTCTGCCCCTGACAA
TGCTTGAGACCTTCTGAATGCATTCTGTTGCTGCTGCATGTTCCTCTACCTG
ATGATAGTCACTGCTGCCCATAACTGGTAAGATTTAATTCACCCTTGGGAA
AGACCACATCTACCTTTTTACTCAGGGCAAGTAAATAATTTGGGTGCTGG
ACCACTTGTCATGGGAACTCATATTCTGACCATTGCTGTTATCACTAGCTG
CCCACCTATTGCAGAGAGGTAGCAGCAGTCTGGGGAGGAAGACTTCAGAT
TTTGAGAAATAATTTAGGGGAAGTTAAACTGATCGTTTTGAAATGTGAGT
ACTGTTTAAATGTTTTAGTATAGTTATAAGGATGTATATCTGGTAAAGAAA
AATTAAAGCACTAAAAAAAGGGAATGTTGTATTTAATAAGAGATTTACTT
TTGTTACTTCCTCATTTAACAGAAAAATCCAGCATGTACTAATTTGTTTAC
AGGTGCTTTTCAGTGGGAAGAATTGGAAGAAGATATCAGGAAGAAGTTG

AAAGATTCTGGGGATGTTTCAAATGTAATCGAGAAAGTTAAATGCATTTT
AAAAACACCAGGAAAGGTAAGTTGATTTGTTGTAAGTTAGTTTACTTTGA
CCCTCTGAATTATAACTCACATACTATGATGAATATAAAATTGAGTCGTTA
GAAGAGTCAAATACTGTATAAATGTAAATGTTTTCCTTAAGTATATTTTAC
CATTTAAATATTTAAAACATAAACATGGGTTATTTGTATCTTAGATTGTGT
GTGTGTGTGTGTGTGTGTGTGAGACAGAGACCTGTGTTGCCCAGGTGG
GAGTGCGTTGGCATGATCTTGGCTTACTGCAGCCTCCACCTCCTGGGCTCA
AGTGATATCTCCCACCTCAGCCTCCCAAGGAGCTGGGACTATAGATATGC
ATCACCATACCTGGCTAATTTTTAAATTTTTTGTAGAGACGTGGTTTTTCC
ATGTTGCCCAGGCTAGTCTCAAACTCCTGGGCTCAAGCGATCTGCCCACC
GTAGCCTCCCAAGGTGCTGGGATTACAGGCATGAGCCACCACACTCGGCC
CTATCTTAGACTTTAATTTATGTAAGAATTAAAATACATGTGAGTCATGTC
TTTAAAATGCTCGTTTGCGAATTCTGCCTTCTGAAACATTCCCCTGGAGCT
CTGGGAATACACAATCTATATCATGAATAAAAGGCTGTTTAATTTGTCCA
GGTACAGTTAAGGTTATTAGAGAGGGTGCCTTCTCACTCCTCACAATTTAA
GTCTCAAATAGTTTTTGAGAGGAAGGCAGTTTTGAATGTCCCTTTTTCAAG
TGTTCTGAGAAATCTATTCAACGAGGTGGACAGTTAAGGATGATCTCATC
AATTATAGGTTCAAAATTATAAATTAGATTCAAGTTTATAAAAACAGACT
TTGTATATTGCCATAGACTGGTGCACATTAAAACTCTAGTAACTTGGTATT
GTGTAGTCTGTAAGGGCTTTAAAAAACATTTCAAAACTTTAAGACATTAC
ACAATTTAGCCAGTGTATTTTTTTGTCTGATTTTGTGATAAACAAATTGGC
TGCAGTAGATAAGTAAGGTAAGTTATATGCAATACTTAGCGGAGTAAAGG
TATATATTAACTATAAGAAAAATGATGATTCCTTTAATTTTTTAATATAGA
TGACTTTTCTGTTTTCTAAAACCTGTTAACATTTTAAAGTTAATGTTTTACA
AAAATGAAACTGTACTAGCTTTTAGTGAAGATTCTTGATTGTTCTTTAGTT
GAGAAAATAGTCTTTTTTGTAAACAAAGTAACGTGGAATTAAGGGGTAAG
ATTTTTTTTTTTTTAGTAACACATTGAAGCAGGTATACAGAAGCTGTCCAG
TTTTACAAGTATATGGCAGGATGGTAGCTCTTGCAGTCTAATTCAACTAAA
TAATCATTTGTAAAAATTTGCACATATGCCTATATTCTCGTTTTCAAAACA
GTTCATCTAGTTGTCTTGATTAACTTGTTTACGGACCAGTGAAAATGTACA
TCACGTACATTTGTGTTCTCTCAAACTTCTGCTCTCCCATTTTTTTCAAATT
CAAAACAGAATTGATGAGGGAATCTGTTTATAGATTCTCTCCCTCTCTCTC
CAGTTCTCTCATACATTCATGTGTAATATACATCTATAAAATATTCATAAT
ATGCAAATTCATAACCTTTATATGGCTGACAAGTACAGAAATCTTGCCTTT
TCTTCTAACCCTAAACCTTTTCTTGAGTCTTGATTCGGAACACATTCCTTTG
AATACTGAGAATAAAATGAATTCTTATTGGGGAAAGGGAAGGCTGGGTG
ATACGATTCCATGGTATTGCGCTGAATTCCCTCCTCTGCACGGGGAGAAA
GAAGAGTTATTTCACACCTTCTTCCTGTGCTTTCCCAGGGTAAGGGGCTGT
CTCATTTACTGAGTACCACACAGGGGTGTAACTCTGAATTGGGACTTGGG
AAATGTAGATTACAATTGTGAATCTGCCACAAATCATCTCTGTGGCTTTGA
GCAAGTCAGGGCTTCCTATAAAATGAGGGGTAAATTGGATGGTTTCTGAA
GCCTCTGCGAGCTCTCCAATTCAGTGACGCTTTTTGTTTTTGTTTTTTGAGA
CGGAGTCTCGCTCTATCGCCCAGGCTGGAATGCAGTGGTGTGATCTCTGCT
CACTGCAAGCTCCGCCTCCCGGGTTCATGCCATTCTCCTGCCTCAGCCTCC

CGAGTAGTTGGGACTACAGGCGCCCGCCACCACACCCAGCTAATGTTTTT
GTATTTTGAGTGGAGACGGGGTTTCACCGTGTTAGCCAGGATGGTCTCGA
TCTCCTGACCTTGTGATCCTCCCGCCTCGGCCTCCCAAAGTGCTGGGATTA
CAGGCATGAGCCACCGTGCCCAGCCCGGTGATGCTTTTTGACTGCTACTCT
CGTTAAGCGTGGCGGTCACTTGACTCCCATGCCCGAATTCCTTCCATGTGT
ATGACGGTTATGTTGTGTATAGGATCTCAGAATGACTTTATAGGACCAGG
ATTTGTATGATCCTGTTTTGCCTCTCTGTTTTGCTTATGGATACCATTCTGG
AATGTTTTCTTCTGCCTTAGGAGGAAGAATAATATCATTTTTCCCCCCTCA
CCGAAGATTTTAAACTTCCCACCATAGTAGATTAGAGTAAGAAGTAGATG
TCATTTATTCTTATTACTGCTGATGTTATATGTTTATTTCTTAGGGATAAAG
CTTTCAGAGTTCCCTAGCTAGGCAAGCAGAGGATTAGCATTCTGACCAGG
GCTCTCAGGCTCTGAGATTCAGCAGGGGGAATGGGTGGAGACGTCAGAAT
TTAAAGTGAATAAAATATGATATTTCAATAAAAATCATAACTAAGTCTTTC
CAAATTAGGGAACAACATAGACTTTAGGCAAGATTGTGGTCCTAATGAAT
CCTAGTATTTAAAATGACTTATTATTTCATCATCATTTGGGTAACACTACC
TGAAAATATTCAGGCTTGTTTTGTTTATCCACTGTAAATAGACTAAAATTT
AAAAAAAAAAAAAAAAAAGAGAAAGAAAGCAAAGCAAAGAAAAAAGGGT
GGAGGGTATGGAAAGGGAGGCCAGCCCTTGACTTCCAGGAGGGTTGGCA
CACAGGGGATACCTGAAGACCACAGAAAGGATAAAGCACTGAGGTCTGA
GTTAGACAGAGAGGCTGCTCCCGGAGCCACTAGCTCCAGCCAGGCGCGAC
AGGACAGACGAGTTATCAAGGGTGAGAGTCTCCGGAACGCCAGATCAAA
AACCCAGGAAACTCTTTATAGGTTCTAGTGGGCTCTGCTGTGAGTAGGAA
GGACAATTAGGATTATAAGATTGGAGATGAAGATGGAGGTGAACCAGCT
GGGCCCGGTGGCTCACACCTGTAATCCTAGCATTTTGGGAGGTCAAGGTA
GGCGGATTGCCTGAGGTTAAGAGTTTGAGACCAGCCTGGGAAAAATGGCA
AAACCCTGTCTCTACAAAAAATCAGCTGGGCGTGGTGGTGCATGCCTGTA
GTCCCAGCTTCTCGGGAGGCTGAGGCACGAGAAGCACTTGAGCCTGGGAG
GTGGAGGTTGCAGTGAGCCAATATCGCACCATTGCACTCTAGCCTGGGCC
ACAGAGCAAGACTCCATCTCAAAAAAGATGGAGGTGAACCACTTCATTAC
TCTACGTTATTTTTTTAACTGTTCACTCCTTTAAGAGAAAATGTTAAGCTA
GTAAGTAGAAGAAGGATATGGTGTGATAAGGGAAAGTTGTATTTTTCTTT
CTTTTTTTTTTTTGGAGACAGGGTTTCACTCTGTTGCCCAGGCTGGAGTGC
AGTGGCATAATCTCAGTTTACTGCAGCCTCTGCCCCCTGGGTTCAAGTGAT
TCTACTGCCTCAGCCTCCCAAGTAGCTGGTATTACAGGCACATGCCACCAT
GTCCAGCTAATTTTTGTATTTTCAGTAGAAACAGGGTTTTGCTATGTTGGC
CAGGCTGGTCTCAAACTCCAGACCTCAAGTGCCCACCTTGGCCTCCAAAG
TGCGGGATTATAGGCATGAGCCACTGCACCTGGCCAAAAAATGTATTTTT
CTTAATTACAAATCAAGGACTGTCTTAATGAACAAATTAGGTTGTACATGT
AAACATATTTTAGTAGGCATTTGTATGGAGTTGGACTACTGTTGTCTGTTT
CTCAACAAAGACTCATAGGGTTTCAGGACCTTGCAGCTTCTCAGCAATGG
AGCTGAGTTTTCACTTAGGCAATTTGTTTACTGCTGTGAATGCCAAAGAGA
GCAGTGTTGTTCACTGACAGGCAGCCCATACAGTTCTTAAAGGTAAGTTC
CTTTGAAACCTGTGATCAGAGATGTCATAGAGTAGGGAATCATATTTCTTT
TGAGATTTAGCATAATTTTCCAAATTCCTAGCCTAGCCTATATGTTATCAA

AGCATGTTAAACAACCTTTCCCTGTATTTTTGTTTTCCCTTATAGATTAATT
GCCTAAGGAATTGCAAAACCTATCAAGCCAGAAAACCTTTGTGGTTTTAT
AACACTTCCCTCAAGTTTTTCCTTAATAAGCCGATGCTTGCCGATGTTGTC
TTCGAAATTCAAGGTACGGATCAACTTTTACAAAGTTTATGATTCATTTTT
ATTTCTTGTTTTCTTTCCTTGCTTTCTACTTCAGGTTTGAATGTTTGGTGTG
AAGGAATGATTATTCCCTACTGAGGTATGTAGATTAATACTACCTTAATGT
CAACAAAATCTTCCAGGTAAATAGGAAGTATTTTATTAAAATGATAAAAT
AAATTTATATTTCCCTTAGTATTTTTTCCTTTGCCATATGCTTCATCAACTT
TTAGGGCCTAGCAAGGGATGGGGTGATCTATAATATTTTATTATATATTTT
TGTGTGTATTTCTTTAAGATCCTAGTGTTTGCTGCATCTGGAGCTAACCCC
CTTAAAATTATTTTATCTCTAGCTTATTTTATCCAGCTCTCTTCCTTTTTATT
TGAGCAAAAGGAATTGACCATTATTTTATTCAGCTGTCTTCTGTTTCTTTTG
AGCA

Note:

1. **5kb intron upstream of RHOBTB3 Exon6 have 3 Alu elements and the sequences are marked with yellow background.**
2. **5kb intron downstream of RHOBTB3 Exon7 have 3 Alu element and the sequence is marked with gray background.**
3. **circRHOBTB3 sequence region (Exon6-Exon7) with green background.**
4. **FUS binding sites are marked with red background.**

>hg19_rmsk_AluY range = chr5:95088556-95088856 5'pad = 0 3'pad = 0 strand = + repeatMasking = none
CCGGGCGCGGTGGCTCACACCTGTAATCCCAGCACTTTGGGAGGCCGAGG
TGGGAGGATCACAAGGTCAGGAGATCGAGACCATCCTGGCTAACACGGT
GAAACCCCGTCTCCACTAAAAATACAAAAAAATTAGCCGGGTGAGGTGGC
AGGCGCCTGTAGTCCCAGCTACTCGGGAGGCTGAGGCAGGAGAATGACGT
GAACCCAGGAGGCGGAGCTTGCAGTGAGCAGAGATTGTGCCACTGCACTG
CACTCTGGCCTGGGCGACAGAGCAAGACTCTGTCTCAAAAAAAAAAAAG
AAA

>hg19_rmsk_AluSx range = chr5:95089534-95089781 5'pad = 0 3'pad = 0 strand = + repeatMasking = none
CCAGGTATGGTGGCTCATGCCTATAATTCCAGCACTTTGGGAGGCCAAGG
TGGGCAGATCACCAGGGGTCAGGAGTTCGAGACCAGTCTGGCCAACATGG
TGAAACCCCGTTTCTACTAAAAATACAAAAATTAGCCGGGCATGATGGCG
ACGCCTGTAGTCCCAGCTACTCGGAAGGCTGAGGCAGAGCTGCATGAACC

AGGGTGGCAGAGGTTGCAGTGAGCCAAGATCATACCACTGCACTCCAG

>hg19_rmsk_AluJb range = chr5:95099535-95099813 5'pad = 0 3'pad = 0 strand = - repeatMasking = none
GGCCGAGTGTGGTGGCTCATGCCTGTAATCCCAGCACCTTGGGAGGCTAC
GGTGGGCAGATCGCTTGAGCCCAGGAGTTTGAGACTAGCCTGGGCAACAT
GGAAAAACCACGTCTCTACAAAAAATTTAAAAATTAGCCAGGTATGGTGA
TGCATATCTATAGTCCCAGCTCCTTGGGAGGCTGAGGTGGGAGATATCAC
TTGAGCCCAGGAGGTGGAGGCTGCAGTAAGCCAAGATCATGCCAACGCA
CTCCCACCTGGGCAACACAGGTCTCTGTCT

>hg19_rmsk_AluY range = chr5:95101366-95101665 5'pad = 0 3'pad = 0 strand = - repeatMasking = none
GGCTGGGCACGGTGGCTCATGCCTGTAATCCCAGCACTTTGGGAGGCCGA
GGCGGGAGGATCACAAGGTCAGGAGATCGAGACCATCCTGGCTAACACG
GTGAAACCCCGTCTCCACTCAAAATACAAAAACATTAGCTGGGTGTGGTG
GCGGGCGCCTGTAGTCCCAACTACTCGGGAGGCTGAGGCAGGAGAATGG
CATGAACCCGGGAGGCGGAGCTTGCAGTGAGCAGAGATCACACCACTGC
ATTCCAGCCTGGGCGATAGAGCGAGACTCCGTCTCAAAAAACAAAAACA
AAAA

AluY Matches AluY:

Range 1: 1 to 300 Graphics ▼ Next Match ▲ Previous Match

NW Score	Identities	Gaps	Strand
439	272/305(89%)	5/305(1%)	Plus/Plus

```
Query  1      CCGGGCGCGGTGGCTCACACCTGTAATCCCAGCACTTTGGGAGGCCGAGGTGGGAGGA  58
                | |||| ||||||||||  ||||||||||||||||||||||||||||||| |||||||
Sbjct  1    GGCTGGGCACGGTGGCTCATGCCTGTAATCCCAGCACTTTGGGAGGCCGAGGCGGGAGGA  60

Query  59   TCACAAGGTCAGGAGATCGAGACCATCCTGGCTAACACGGTGAAACCCCGTCTCCACTAA  118
            |||||||||||||||||||||||||||||||||||||||||||||||||||||||||| |
Sbjct  61   TCACAAGGTCAGGAGATCGAGACCATCCTGGCTAACACGGTGAAACCCCGTCTCCACTCA  120

Query  119  AAATACAAAAAAATTAGCCGGGTGAGGTGGCAGGCGCCTGTAGTCCCAGCTACTCGGGAG  178
            ||||||||||| |||||| ||||| |||||| |||||||||||||||| |||||||||||
Sbjct  121  AAATACAAAAACATTAGCTGGGTGTGGTGGCGGGCGCCTGTAGTCCCAACTACTCGGGAG  180

Query  179  GCTGAGGCAGGAGAATGACGTGAACCCAGGAGGCGGAGCTTGCAGTGAGCAGAGATTGTG  238
            ||||||||||||||||| | ||||||| ||||||||||||||||||||||||||||    
Sbjct  181  GCTGAGGCAGGAGAATGGCATGAACCCGGGAGGCGGAGCTTGCAGTGAGCAGAGAT----  236

Query  239  CCACTGCACTGCACTCTGGCCTGGGCGACAGAGCAAGACTCTGTCTCAAAAAAAAAAAAG  298
             |||  ||||||| ||  |||||||||| ||||| |||||| ||||||||||| ||||| 
Sbjct  237  -CACACCACTGCATTCCAGCCTGGGCGATAGAGCGAGACTCCGTCTCAAAAAACAAAAAC  295

Query  299  AAA    301
            |||
Sbjct  296  AAAAA  300
```

AluY Matches AluJb:

Range 1: 1 to 279 Graphics ▼ Next Match ▲ Previous Match

NW Score	Identities	Gaps	Strand
137	215/308(70%)	34/308(11%)	Plus/Plus

```
Query  1      CCGGGCGCGGTGGCTCACACCTGTAATCCCAGCACTTTGGGAGGCCGAGGTGGGAGGA  58
                ||| | | |||||||||  |||||||||||||||| |||||||||   ||||||  ||
Sbjct  1    GGCCGAGTGTGGTGGCTCATGCCTGTAATCCCAGCACCTTGGGAGGCTACGGTGGGCAGA  60

Query  59   TCACA--AGGTCAGGAGATCGAGACCATCCTGGCTAACACGGTGAAACCCCGTCTCCACT  116
            || |   ||  |||||| | ||||| | |||||  |||| ||  ||||| |||||| ||
Sbjct  61   TCGCTTGAGCCCAGGAGTTTGAGACTAGCCTGGGCAACATGGAAAAACCACGTCTCTACA  120

Query  117  AAAAATACAAAAAAATTAGCCGGGTGAGGTG--GCAGGCGCCTGTAGTCCCAGCTACTCG  174
            ||||||  |||||  |||||| |||  ||||  |||     || ||||||||||| || |
Sbjct  121  AAAAATTTAAAAA--TTAGCCAGGTATGGTGATGCATAT--CTATAGTCCCAGCTCCTTG  176

Query  175  GGAGGCTGAGGCAGGAGA-ATGACGTGAACCCAGGAGGCGGAGCTTGCAGTGAGCAGAGA  233
            |||||||||||  ||||| || || ||| ||||||||| ||||  |||||| |||  |||
Sbjct  177  GGAGGCTGAGGTGGGAGATATCACTTGAGCCCAGGAGGTGGAGGCTGCAGTAAGCCAAGA  236

Query  234  TTGTGCCACTGCACTGCACTCTGGCCTGGGCGACAGAGCAAGACTCTGTCTCAAAAAAAA  293
            |  |||||    || ||||||   ||||||| ||| ||   | ||||||||
Sbjct  237  TCATGCCA----AC-GCACTCCCACCTGGGCAACACAG---GTCTCTGTCT          279

Query  294  AAAAGAAA   301
```

AluSx Matches AluJb:

Range 1: 1 to 279 Graphics ▼ Next Match ▲ Previous Match

NW Score	Identities	Gaps	Strand
139	196/279(70%)	19/279(6%)	Plus/Plus

```
Query  1      CCAGGTATGGTGGCTCATGCCTATAATTCCAGCACTTTGGGAGGCCAAGGTGGGCAGA  58
              ||  || ||||||||||||||| |||| ||||||| ||||||||| | ||||||||||
Sbjct  1    GGCCGAGTGTGGTGGCTCATGCCTGTAATCCCAGCACCTTGGGAGGCTACGGTGGGCAGA  60

Query  59   TCACCAGGGGTCAGGAGTTCGAGACCAGTCTGGCCAACATGGTGAAACCCCGTTTCTACT  118
            || |  | |  |||||||| ||||| || |||| ||||||||  ||||| ||| |||||
Sbjct  61   TCGCTTGAGCCCAGGAGTTTGAGACTAGCCTGGGCAACATGGAAAAACCACGTCTCTACA  120

Query  119  AAAAATACAAAAATTAGCCGGGCATGATGGCG-ACGCCTGTAGTCCCAGCTACTCGGAAG  177
            ||||||  ||||||||||| || ||| ||  | |   || ||||||||||| || || ||
Sbjct  121  AAAAATTTAAAAATTAGCCAGGTATGGTGATGCATATCTATAGTCCCAGCTCCTTGGGAG  180

Query  178  GCTGAGGCAG-AGCTG-CA--TGAACCAGGGTGGCAGAGGTTGCAGTGAGCCAAGATCAT  233
            |||||||  | || |  ||  ||| ||  || ||  |||| |||||| ||||||||||||
Sbjct  181  GCTGAGGTGGGAGATATCACTTGAGCCCAGGAGGTGGAGGCTGCAGTAAGCCAAGATCAT  240

Query  234  ACCACTGCACTCC--------------AG           248
             |||  |||||||              ||
Sbjct  241  GCCAACGCACTCCCACCTGGGCAACACAGGTCTCTGTCT  279
```

AluSx Matches AluY:

Range 1: 1 to 300 Graphics   Next Match Previous Match

NW Score	Identities	Gaps	Strand
165	211/302(70%)	8/302(2%)	Plus/Plus

```
Query  1      CCAGGTATGGTGGCTCATGCCTATAATTCCAGCACTTTGGGAGGCCAAGGTGGGCAGA  58
                |  || | |||||||||||||| |||| ||||||||||||||||||| |||  |||  ||
Sbjct  1    GGCTGGGCACGGTGGCTCATGCCTGTAATCCCAGCACTTTGGGAGGCCGAGGCGGGAGGA  60

Query  59   TCACCAGGGGTCAGGAGTTCGAGACCAGTCTGGCCAACATGGTGAAACCCCGTTTCTACT  118
            |||| |||  ||||||| |||||||||  ||||| |||| ||||||||||||| || |||
Sbjct  61   TCACAAGG--TCAGGAGATCGAGACCATCCTGGCTAACACGGTGAAACCCCGTCTCCACT  118

Query  119  AAAAATACAAAAA--TTAGCCGGGCATGATGGCGA-CGCCTGTAGTCCCAGCTACTCGGA  175
             ||||||||||||  ||||| |||  || |||||  |||||||||||||| |||||||| 
Sbjct  119  CAAAATACAAAAACATTAGCTGGGTGTGGTGGCGGGCGCCTGTAGTCCCAACTACTCGGG  178

Query  176  AGGCTGAGGCAG-AGC-TG-CATGAACCAGGGTGGCAGAGGTTGCAGTGAGCCAAGATCA  232
            |||||||||||| ||  || |||||||| ||| ||| ||| |||||||||||  ||||||
Sbjct  179  AGGCTGAGGCAGGAGAATGGCATGAACCCGGGAGGCGGAGCTTGCAGTGAGCAGAGATCA  238

Query  233  TACCACTGCACTCCAG                                             248
             ||||||||| |||||
Sbjct  239  CACCACTGCATTCCAGCCTGGGCGATAGAGCGAGACTCCGTCTCAAAAAACAAAAACAAA  298

Sbjct  299  AA  300
```
